# Effects of brain-computer interface-based rehabilitation on lower limb function and activities of daily living after stroke: a systematic review and meta-analysis

**DOI:** 10.3389/fneur.2026.1746958

**Published:** 2026-02-25

**Authors:** Changshuo Liu, Jiaxu Han, Yuhui Wang, Xinyun Liang, Xianguo Meng

**Affiliations:** 1 School of Sports Medicine and Rehabilitation, Shandong First Medical University (Shandong Academy of Medical Sciences), Taian, China; 2 Department of Rehabilitation Medicine, The First Affiliated Hospital of Shandong First Medical University & Shandong Provincial Qianfoshan Hospital, Jinan, China

**Keywords:** activities ofdaily living, brain-computer interfaces, lower limb, meta-analysis, motor function, stroke

## Abstract

**Background:**

Lower limb motor dysfunction is a common sequela of stroke that significantly impacts patients' walking safety and independence in daily living. Although brain-computer interface (BCI) technology has demonstrated efficacy in upper limb rehabilitation, its effects on lower limb recovery have not yet been systematically evaluated.

**Methods:**

A systematic literature search was conducted across seven databases (PubMed, Web of Science, Embase, China National Knowledge Infrastructure, SinoMed, VIP Database, and Wanfang Data.) to identify studies investigating BCI for post-stroke lower limb dysfunction, encompassing records published up to September 2025. All statistical analyses were performed using Review Manager software (version 5.4.1).

**Results:**

Thirteen studies involving 582 participants were included. BCI training significantly improved the scores of Fugl-Meyer Assessment for Lower Extremity (FMA-LE, *MD* = 2.67, 95%CI: 2.31–3.03, *P* < 0.00001, *I*^2^ = 0%), Berg Balance Scale (BBS, *MD* = 7.04, 95%CI: 3.14–10.94, *P* = 0.0004), and Modified Barthel Index (MBI, *MD* = 6.72, 95%CI: 1.74–11.69, P = 0.008). Furthermore, a single study reported significant improvement in functional mobility measured by the Timed Up and Go Test (TUGT). Subgroup analysis for activities of daily living MBI showed that a cumulative training time of ≥ 500 min was associated with greater improvement.

**Conclusion:**

BCI-based training is an effective approach for improving lower limb recovery after stroke, demonstrating benefits in motor function, balance, and functional mobility. While evidence for certain outcomes remains limited, the dose-dependent effect on daily living activities underscores the importance of sufficient training duration. Future research should validate these findings and clarify effects across a broader range of functional measures.

**Systematic review registration:**

https://www.crd.york.ac.uk/PROSPERO/view/CRD420251150558, identifier: CRD420251150558.

## Introduction

As a leading cause of long-term adult disability worldwide, stroke results in functional impairment for approximately 90% of survivors, with severe disability affecting about 40% (1). Lower limb motor dysfunction, particularly in motor control and balance, is among one of the most common and disabling sequelae, significantly compromising patients' capacity for independent daily living and imposing a substantial burden on individuals and healthcare systems. Consequently, regaining lower limb motor function and functional independence represents a primary rehabilitation goal for most stroke patients (2–5).

While conventional rehabilitation approaches are fundamental, they often struggle with limitations when it comes to promoting sufficient neuroplasticity for optimal rehabilitative outcomes. This has catalyzed the exploration of novel technologies, among which brain-computer interface (BCI) has emerged as a particularly promising strategy (6, 7). BCIs are systems that translate or decode brain activity signals into commands, allowing for direct control of external devices without relying on peripheral nerves or muscles. Based on the method of signal acquisition, they are broadly categorized into invasive and non-invasive types (8). The distinctive strength of BCI lies in its core mechanism—a direct “central–peripheral–central” closed loop (9). This system decodes motor intention from cortical signals to drive peripheral devices (e.g., exoskeletons, functional electrical stimulation) in real time, thereby bridging the disrupted pathway between intention and action. By delivering feedback that is timed with precision and contingent on intention, this process establishes an optimal setting for use-dependent neuroplasticity, differing fundamentally from conventional therapies that lean more on physical guidance (10–12). The well-established efficacy of BCI in upper limb rehabilitation, as confirmed by a recent meta-analysis, underscores its therapeutic potential (13–16).

However, despite growing interest in applying BCI to lower limb recovery, the current evidence base remains unsystematic and has not been quantitatively pooled. The overall efficacy of BCI for lower limb motor function and daily living activities thus remains unclear, and guidance on its optimal application is lacking.

Therefore, to address this critical evidence gap, this study will conduct the first systematic review and meta-analysis to definitively establish the efficacy of BCI-based training for improving lower limb motor function and activities of daily living after stroke.

## Methods

We rigorously adhered to established methodological standards for systematic reviews. Our conduct was guided by the Cochrane Handbook, while our reporting followed the PRISMA 2020 statement (17).

### Search strategy

We systematically searched the literature published up to September 11, 2025, across three English databases (PubMed, Web of Science, Embase) and four Chinese databases (China National Knowledge Infrastructure, SinoMed, VIP Database, and Wanfang Data). The search strategy utilized a combination of Medical Subject Headings (MeSH) and free-text terms for the core concepts of “stroke” and “brain-computer interfaces”. The search was restricted to randomized controlled trials (RCTs) published in Chinese or English. The complete search strategy for PubMed is provided in the Supplementary Table 1.

### Eligibility criteria and study selection

The inclusion and exclusion criteria were formulated *a priori* to identify studies that directly evaluated the effect of BCI-based training on lower limb recovery after stroke. Inclusion criteria were as follows: (1) Randomized controlled trials (RCTs). (2) Adult patients (age ≥ 18 years) diagnosed with stroke, confirmed by computed tomography or magnetic resonance imaging, with lower limb motor dysfunction. (3) The experimental group received BCI-based training, which could be combined with conventional rehabilitation. (4) The control group received conventional rehabilitation. (5) Studies had to report at least one of the following outcome measures related to lower limb motor function or activities of daily living: the Fugl-Meyer Assessment for Lower Extremity (FMA-LE), the Fugl-Meyer Assessment for Balance (FMA-B), Time UP and GO Test (TUGT), the Berg Balance Scale (BBS), 6-min Walk Distance (6MWD), or the Modified Barthel Index (MBI). The FMA-LE was prespecified as the primary outcome for meta-analysis given its widespread use and validation for assessing lower-limb motor recovery after stroke. Exclusion criteria included: (1) Duplicate publications. (2) Non-clinical studies (e.g., reviews, meta-analyses, animal studies, case reports). (3) Full-text unavailable after exhaustive search. Two reviewers (CS and JX) independently assessed the studies for inclusion, with any discrepancies resolved by consensus.

### Data extraction

Data extraction was performed by two independent reviewers (CS and JX). The following data were extracted from each included study: first author's name, year of publication, participant characteristics, detailed descriptions of both experimental and control interventions, specific BCI paradigms, outcome measures, and follow-up assessments when available. When studies reported mean change scores and standard deviations, these values were extracted directly. When change scores were not explicitly provided, they were calculated using the following formulas based on the Cochrane Handbook for Systematic Reviews of Interventions (18). In cases where studies reported median and interquartile range, these values were converted to mean and SD estimates using established transformation methods: mean ≈ median and standard deviation ≈ IQR × 1.35. Any divergences were settled through deliberation, and unsettled matters were assessed and resolved by a third reviewer (YH) in order to achieve final consensus.


$$\begin{array}{l} {Mean}_{change}\;=\;{Mean}_{final}-{Mean}_{baseline}\;;\\ SD_{change}\;=\;\sqrt{SD_{baseline}^{2}+SD_{final}^{2}-\left(2\times Corr\times {SD}_{baseline}\times {SD}_{final}\right)} \end{array}$$

### Quality assessment

Two reviewers (CS and JX) independently assessed the methodological quality and risk of bias of the included studies using the Physiotherapy Evidence Database (PEDro) scale. This validated tool, widely used in systematic reviews, evaluates clinical trials through 11 criteria encompassing randomization, allocation concealment, blinding, dropout rates, intention-to-treat analysis, and statistical reporting (19). The first item is related to external validity and is not included in the total score. The remaining 10 items are each scored 1 point if clearly satisfied, yielding a total score ranging from 0 to 10. Based on this score, studies were classified as follows: 9–10 (excellent), 6–8 (good), 4–5 (fair), and below 4 (poor).

### Statistical analysis

#### Effect size calculation

Lower limb motor impairment, balance function, and activities of daily living were the focused outcomes in this meta-analysis. The FMA-LE is the most frequently utilized and internationally recognized clinical measurement for assessing motor recovery of the lower limbs in stroke patients, and it was adopted as the primary outcome measure in this study (20). Assessments of balance function included the BBS (21). The MBI was used to assess activities of daily living (22). MD in change scores and 95% CI were calculated for each statistical analysis to assess the efficacy of BCI-based training. A *P*-value of less than 0.05 was considered statistically significant. All data synthesis and analyses were performed using RevMan 5.4.1.

#### Heterogeneity analysis

Statistical heterogeneity among the included studies was quantitatively assessed using the I^2^ statistic and the Chi^2^ test. The I^2^ statistic was interpreted as follows: 0%−40% indicated negligible heterogeneity, 30%−60% moderate heterogeneity, 50%−90% substantial heterogeneity, and 75%−100% considerable heterogeneity (23). A *P*-value < 0.10 for the Chi^2^ test was considered statistically significant for heterogeneity. Based on this assessment, a fixed-effect model was applied when heterogeneity was low (*I*^2^ ≤ 50% and *P* > 0.10), whereas a random-effects model was adopted in cases of substantial heterogeneity (*I*^2^ > 50% and/or *P* ≤ 0.10). To explore potential sources of heterogeneity and verify the robustness of the results, we conducted subgroup and sensitivity analyses.

#### Subgroup analysis

Subgroup analyses were stratified by the number of BCI training treatment sessions (<20 vs. ≥ 20), intervention duration (<4 weeks vs. ≥ 4 weeks), cumulative treatment time (<500 min vs. ≥500 min), mean patient age (≤55 years vs. >55 years), disease course (≤90 days vs. >90 days) and BCI paradigm (BCI+Pedaling vs. BCI+FES). These subgroup analyses were conducted for all outcome measures, including the FMA-LE, BBS, and MBI.

#### Publication bias

A funnel plot was constructed to evaluate potential publication bias, given that the analysis comprised more than ten studies. A symmetric distribution of effect estimates around the overall mean would indicate a low risk of publication bias, while any observed asymmetry could be attributed to random sampling variation.

## Results

### Search results

**Figure 1:**
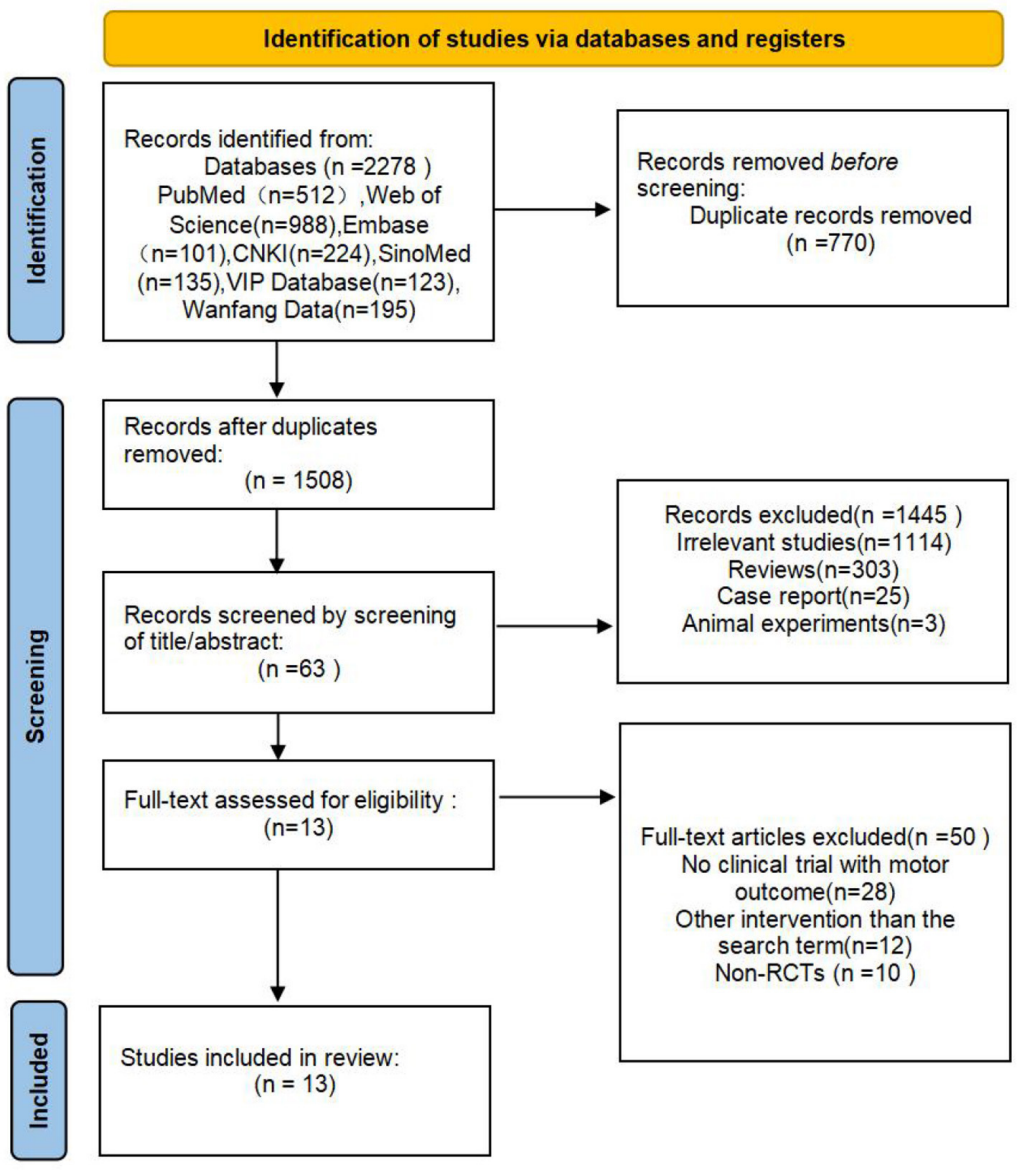
PRISMA flow chart of study selection.

The search strategy yielded 2,278 articles from various sources: 512 from PubMed, 988 from Web of Science, 101 from Embase, 224 from China National Knowledge Infrastructure, 135 from SinoMed, 123 from VIP Database, 195 from Wanfang Data. After removing duplicates and, the titles and abstracts of 1,508 articles were screened for possible inclusion. Subsequently, 63 articles were assessed for eligibility through full-text screening. Finally, 13 articles were included in the meta-analysis (24–36). The flowchart of the search strategy and selection steps is presented in Figure 1.

Table 1 summarizes the baseline characteristics of the included studies, including details of the participants, interventions, and outcome measures. This systematic review and meta-analysis included 13 randomized controlled trials (RCTs), all conducted in China, with 12 published in Chinese and 1 in English. A total of 582 stroke patients were involved, with the mean age ranging approximately from 44 to 66 years across the studies. The experimental interventions consisted of BCI combined with conventional rehabilitation or other physical therapies (e.g., robotics, exoskeletons, rTMS), while the control groups received the corresponding therapy without the BCI component. Few studies clearly reported the specific disease stage of the included stroke patients. Moreover, all outcome measures were assessed using validated instruments and standardized methods. The included studies demonstrated a moderate to good level of methodological quality (Table 2).

**Table 1:** Characteristics of including studies.

**Author, year**	**Study design**	**Publish language**	**Place of study**	**No.of participants (T/C)**	Age (year)		Intervention		Disease Course		**Frequency and Duration of BCI treatment**	**Outcome**
					**BCI**	**Control**	**BCI**	**Control**	**BCI**	**Control**		
Fang,2018	RCT	Chinese	China	40 (20/20)	61.35 ± 9.94	64.45 ± 8.94	BCI+Pedaling, CR	CR	24.25 ± 6.28	25.70 ± 5.87	60 min per time and fifth per week for 4 weeks,20 times in total	FMA-LE
Gao,2023	RCT	Chinese	China	64 (32/32)	54.63 ± 11.11	53.94 ± 11.48	BCI+Pedaling, CR	CR	90.28 ± 76.48	107.06 ± 96.82	30 min per day and sixth per week	FMA-LE, MBI
Han,2025	RCT	Chinese	China	28 (14/14)	44.85 ± 14.25	44.31 ± 11.05	BCI+Pedaling, CR	CR	NR	NR	10 min per group,2 groups per day and fifth per week for 4 weeks	FMA-LE, MBI, BBS
Huang JQ,2024	RCT	Chinese	China	30 (15/15)	66.80 ± 4.41	64.73 ± 4.48	BCI+Pedaling, CR	CR	93.10 ± 40.36	98.60 ± 40.72	20 min per time for 2 weeks	FMA-LE
Huang Y,2024	RCT	Chinese	China	40 (20/20)	57.85 ± 11.79	57.90 ± 12.33	BCI+Pedaling, CR	CR	NR	NR	10 min per group, 2 groups per day and fifth per week for 4 weeks	FMA-LE, BBS
Jin, 2024	RCT	Chinese	China	49 (24/25)	46.98 ± 8.56	49.33 ± 10.51	BCI+Pedaling, CR	CR	NR	NR	30 min per time and for 4 weeks	FMA-LE, FMA-B
Li, 2024	RCT	Chinese	China	73 (36/37)	53.25 ± 11.87	59.00 ± 13.30	BCI+Pedaling, CR	CR	NR	NR	30 min per time and sixth per week for 12 days	FMA-LE
Liu, 2024	RCT	Chinese	China	30 (15/15)	55.00 ± 7.43	55.53 ± 7.30	BCI+FES, CR	CR	27.9 ± 11.83	29.4 ± 10.58	30 min per time and fifth per week and for 4 weeks	FMA-LE, MBI, BBS
Luo, 2023	RCT	Chinese	China	64 (32/32)	NR	NR	BCI+FES, CR	CR	13.72 ± 1.51	15.03 ± 3.40	60 min per time for 2 weeks, 20 times in total	FMA-LE, MBI
Sun, 2023	RCT	Chinese	China	60 (30/30)	53.400 ± 13.34	54.570 ±12.29	BCI+FES, CR	CR	NR	NR	1 time per day and fifth per week for 3 weeks	BBS, FMA-LE, MBI
Tang, 2024	RCT	Chinese	China	40 (20/20)	62.45 ± 10.41	60.95 ± 8.17	BCI+Pedaling, CR	CR	43.60 ± 26.54	46.80 ± 29.51	20 min per time and fifth per week for 4 weeks	FMA-LE, BBS
Wan, 2025	RCT	English	China	30 (14/16)	57.07 ± 11.65	56.25 ± 15.19	BCI+FES, CR	CR	NR	NR	20 min per time and fifth per week for 4 weeks	BBS, TUGT, FMA-LE,
Zhai, 2025	RCT	Chinese	China	34 (17/17)	58.00 ± 12.35	62.06 ± 10.49	BCI + Pedaling, CR	CR	65.76 ± 44.82	59.17 ± 32.88	30 min per time and fifth per week for 2 weeks	FMA-LE, BBS, 6MWD

RCT, randomized controlled trial; T/C, experimental group/control group; BCI, brain-computer interface; CR, conventional rehabilitation; MI, motor imagery; rTMS, repetitive transcranial magnetic stimulation; FMA-LE, Fugl-Meyer Assessment of Lower Extremity; MBI, modified barthel index; BBS, berg balance scale; 6MWD, 6-min walk distance; FMA-B, Fugl-Meyer assessment for balance; TUGT, time UP and GO test.

**Table 2:** PEDro score for methodological quality assessment of including studies.

**Study**	**score**	**Random allocation**	**Concealed allocation**	**Baseline Comparability**	**Blind subjects**	**Blind therapists**	**Blind assessors**	**Adequate follow-up**	**Intention to treat analysis**	**Between-group statistical comparisons**	**Point estimates and variability**
Fang, 2018	7	1	0	1	0	0	1	1	1	1	1
Gao, 2023	8	1	1	1	0	0	1	1	1	1	1
Han, 2025	7	1	1	1	0	0	0	1	1	1	1
Huang JQ, 2024	6	1	0	1	0	0	0	1	1	1	1
Huang Y, 2024	6	1	0	1	0	0	0	1	1	1	1
Jin, 2025	6	1	0	1	0	0	0	1	1	1	1
Li, 2024	7	1	0	1	0	0	1	1	1	1	1
Liu, 2024	5	1	0	1	0	0	0	0	1	1	1
Luo, 2025	5	1	0	1	0	0	0	0	1	1	1
Sun, 2023	6	1	0	1	0	0	0	1	1	1	1
Tang, 2024	7	1	1	1	0	0	0	1	1	1	1
Wan, 2025	5	1	0	1	0	0	0	0	1	1	1
Zhai, 2025	5	1	0	1	0	0	0	0	1	1	1

### Effects on lower-limb motor impairment

#### FMA-LE

**Figure 2:**
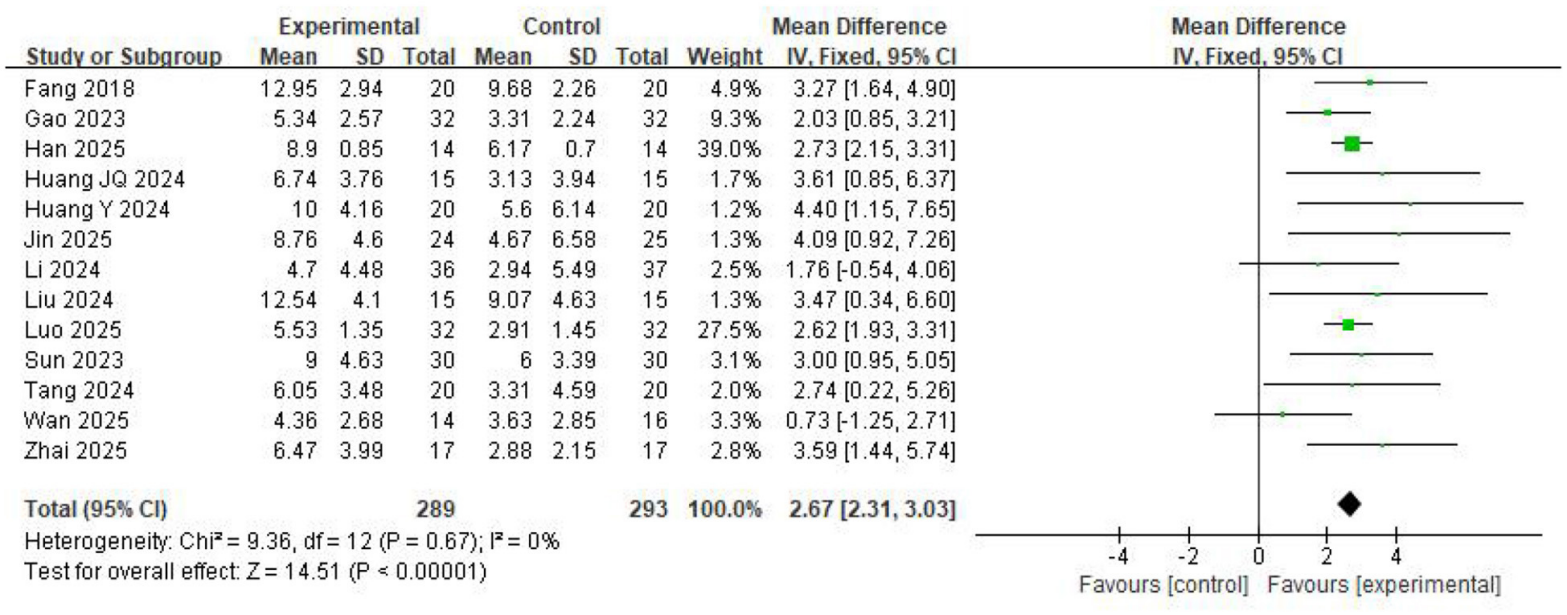
Forest Plot for the FMA-LE.

Thirteen studies involving 582 participants showed that BCI-based training significantly improved FMA-LE scores compared with control group (*MD* = 2.67, 95%CI: 2.31–3.03, *P* < 0.00001) (Figure 2). Heterogeneity analysis revealed negligible heterogeneity among the included studies (*I*^2^ = 0%, *P* = 0.67). The sensitivity analysis demonstrated that the results were stable.

#### BBS

**Figure 3:**
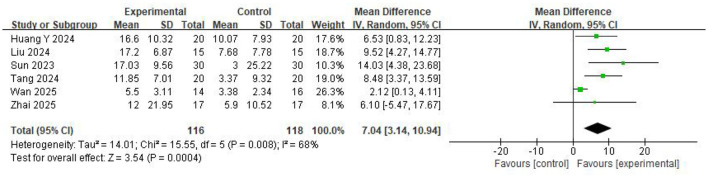
Forest Plot for the BBS.

Six studies involving 234 participants reported that BCI-based training significantly improved BBS scores compared with control group (*MD* = 7.04, 95%CI: 3.14–10.94, *P* = 0.0004) (Figure 3). Heterogeneity assessment indicated substantial heterogeneity across the studies (*I*^2^ = 68%, *P* = 0.008). Leave-one-out sensitivity analysis revealed 1 study contributing most to heterogeneity. The heterogeneity was decreased significantly after excluding Wan et al. (*I*^2^ = 0%, *P* = 0.73) (35).

#### 6MWD, FMA-B and TUGT

Only a single study reported outcomes for 6WMD, FMA-B, and TUGT; therefore, a meta-analysis was not performed, and the results are presented descriptively. Specifically, Gao et al. found no statistically significant between-group difference in 6WMD (*P* = 0.333) (25); Jin et al. reported that while FMA-B scores improved significantly over time, there was no significant difference between groups (29); whereas Wan et al. demonstrated that the improvement in TUGT was significantly greater in the BCI group than in the control group (*P* = 0.038) (35).

### Effects on activities of daily living

**Figure 4:**
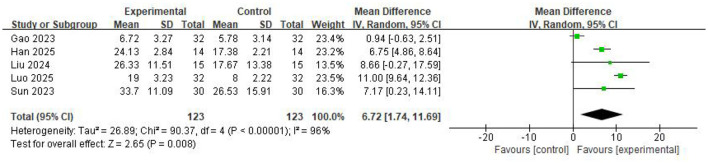
Forest Plot for the MBI.

Five studies involving 246 participants reported that BCI-based training significantly improved MBI scores compared with control group (*MD* = 6.72, 95%CI: 1.74–11.69, *P* = 0.008) (Figure 4). Heterogeneity assessment indicated considerable heterogeneity across the studies (*I*^2^ = 96%, *P* < 0.00001). The leave-one-out sensitivity analysis confirmed that the pooled results were robust, however, substantial heterogeneity persisted.

### Subgroup analysis

#### FMA-LE

To assess the robustness of the pooled results, a series of pre-specified subgroup analyses were performed based on the number of treatment sessions <20 vs. ≥20), intervention duration (<4 weeks vs. ≥4 weeks), cumulative treatment time (<500 min vs. ≥500 min), mean patient age (≤55 years vs. >55 years), disease course (≤90 days vs. >90 days) and BCI paradigm (BCI+Pedaling vs. BCI+FES). As presented in Supplementary Figures 1–6, no statistically significant subgroup differences were observed for any factor. Specifically, the BCI intervention yielded significant and highly comparable improvements in FMA-LE scores across all subgroups—for treatment sessions (*MD* = 2.51 vs. *MD* = 2.70), intervention duration (*MD* = 2.58 vs. *MD* = 2.75), cumulative time (*MD* = 2.54 vs. *MD* = 2.80), patient age (*MD* = 2.64 vs. *MD* = 2.88), disease course (*MD* = 2.81 vs. *MD* = 2.28) and BCI paradigm (*MD* = 2.75 vs. *MD* = 2.51). Furthermore, heterogeneity was negligible in most subgroup comparisons.

#### BBS

The subgroup analyses revealed that the experimental intervention was significantly superior to the control group across all categorizations based on the number of treatment sessions (<20 vs. ≥20), intervention duration (<4 weeks vs. ≥4 weeks), cumulative treatment time (<500 min vs. ≥500 min), mean patient age (≤55 years vs. >55 years) BCI paradigm (BCI+Pedaling vs. BCI+FES) (Supplementary Figures 7–11). However, the tests for subgroup differences yielded *P*-values greater than 0.05 for all factors (*P* = 0.31, *P* = 0.31, *P* = 0.18, and *P* = 0.07, respectively), indicating that none of these variables statistically significantly moderated the intervention effect. It is noteworthy that for the age subgroup, although the between-group difference did not reach the conventional significance level, a trend was observed suggesting a potentially larger benefit in younger patients (<55 years, *MD* = 10.55) compared to older patients (*MD* = 5.11). Similarly, all two BCI paradigms demonstrated significant improvement in BBS scores, with no statistically significant difference in efficacy between them.

#### MBI

Subgroup analyses were conducted to explore potential sources of heterogeneity in the improvement of MBI scores (Supplementary Figures 12–15). The subgroup analysis revealed a statistically significant difference based on cumulative treatment time (*P* = 0.02), with the subgroup receiving more than 500 min (*MD* = 10.95, 95% CI: 9.60–12.29, *I*^2^ = 0%) demonstrating a greater effect than the subgroup receiving less than 500 min (*MD* = 3.82, 95% CI: −1.87–9.51, *I*^2^ = 95%). Subgroup analysis by BCI paradigm revealed a statistically significant difference between groups (*P* = 0.02). The BCI+FES subgroup demonstrated a large and statistically significant improvement in MBI scores (MD = 10.81, 95% CI: 9.49–12.13, *I*^2^ = 0%). In contrast, the BCI+Pedaling subgroup did not show a statistically significant effect compared to control (*MD* = 3.82, 95% CI: −1.87–9.51, *I*^2^ = 95%). In contrast, no significant subgroup differences were found for the number of treatment sessions (*P* = 0.10), although the point estimate was higher in the subgroup with more than 20 sessions (*MD* = 8.90, 95% CI: 5.24–12.56, I^2^ = 84%) compared to fewer than 20 sessions (*MD* = 6.72, 95% CI: −6.41–19.84, *I*^2^^2^ = 66%). Similarly, the analysis based on intervention period showed no significant difference (P = 0.90), with comparable effects between subgroups longer than 4 weeks (*MD* = 6.83, 95% CI: 4.99–8.68, *I*^2^ = 0%) and shorter than 4 weeks (*MD* = 6.32, 95% CI: −1.71–14.35, *I*^2^ = 98%).

### Testing for publication bias

**Figure 5:**
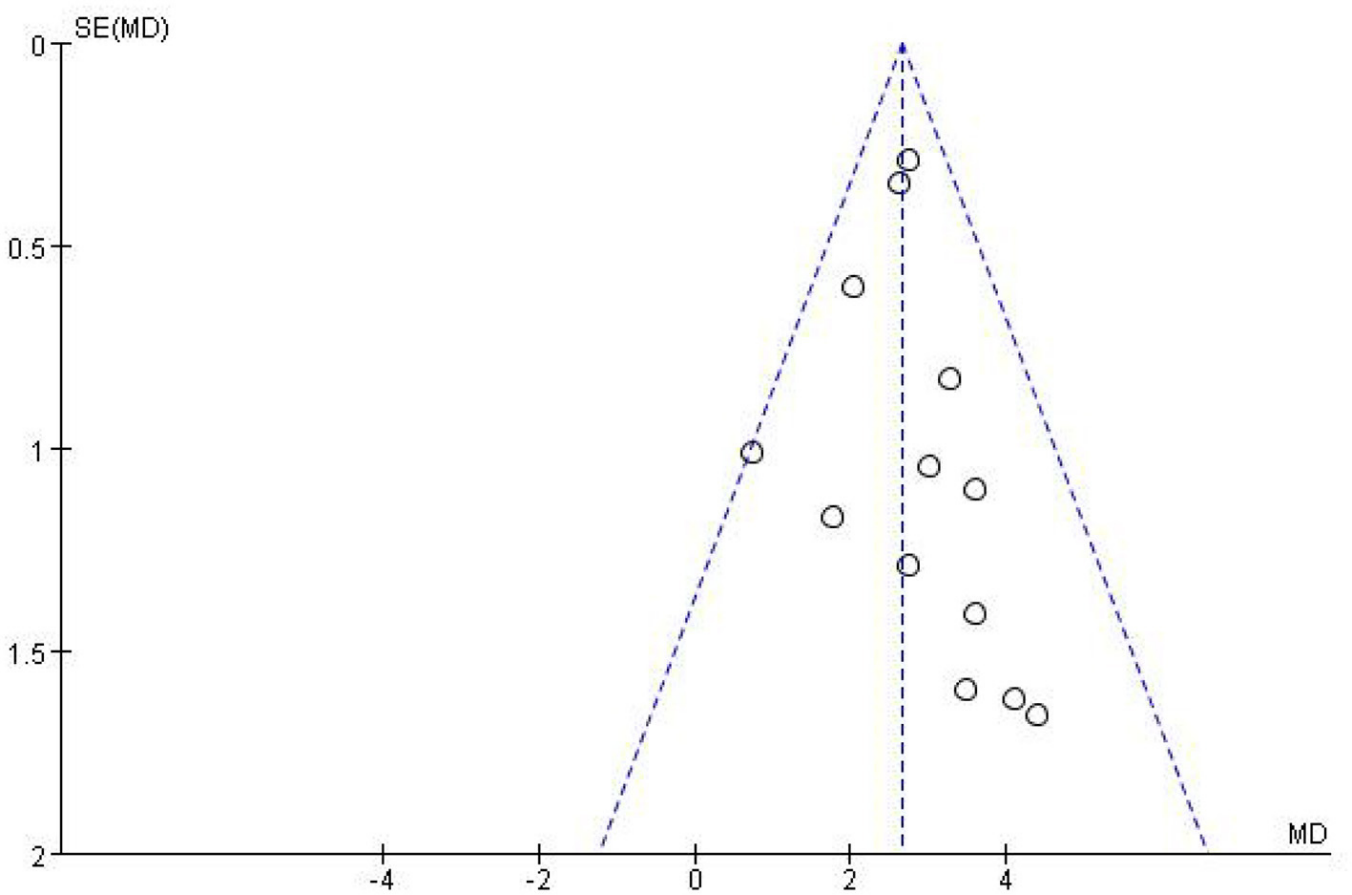
Funnel plot for publication bias.

Visual inspection of the funnel plot for the primary outcome revealed a symmetric pattern with no marked asymmetry (Figure 5). These results suggest a low probability of significant publication bias, though we recognize the limitation of a small number of studies for a conclusive assessment.

### Adverse events

There were no adverse events documented in any of the included studies.

## Discussion

This study comprehensively evaluated the rehabilitative effects of BCI technology on lower limb function in stroke patients through a systematic review and meta-analysis. The pooled findings show that BCI training effectively fosters the recovery of lower limb motor and balance function, which ultimately translates to measurable improvements in activities of daily living. Additional subgroup analyses indicated that the efficacy of BCI differed across various functional domains: while it reliably improved motor and balance function, its impact on activities of daily living displayed a distinct dose-dependent relationship with total intervention time.

### Effects of BCI on lower limb motor function

FMA-LE is an internationally recognized gold standard for assessing the recovery of limb motor function after stroke. This meta-analysis included 13 studies that evaluated the effect of BCI on FMA-LE scores. The pooled results showed that BCI intervention significantly increased patients' FMA-LE scores compared to the control group, with no heterogeneity observed among the studies. This indicates that BCI training is clearly effective in improving lower limb motor impairment in stroke patients. The improvement in lower limb motor function following BCI therapy depends on its task-specific closed-loop modulation that precisely targets the remodeling of cortico-spinal pathways governing gait and postural control (37–40). This mechanistic perspective is corroborated by a recent comprehensive review covering advances in BCI technology from 2023 to 2024, which highlights that BCI systems facilitate cortical–spinal pathway remodeling through intention-driven, closed-loop paradigms, thereby enhancing motor recovery after neurological insults (41). By decoding movement intention from the sensorimotor cortex to trigger exoskeletons or functional electrical stimulation for performing task-oriented actions like weight-bearing and stepping (42), the system establishes direct efferent-afferent coupling (43, 44). This process not only enhances coordinated activation between the primary motor cortex and motor planning regions (45). More critically, delivers precisely timed proprioceptive feedback that is spatially congruent with the intended movement (42, 46, 47). This exact spatiotemporal correspondence is considered pivotal for optimizing the function of central pattern generators at the spinal level and rebalancing interhemispheric inhibition (40, 45, 48), thereby effectively promoting the functional reorganization of neural networks essential for weight-supported standing and coordinated locomotion (49–52).

Balance dysfunction is a common issue after stroke, severely affecting patients‘ transfer ability and walking safety (53). This analysis used the BBS for assessment, and the results showed that BCI training significantly improved patients' balance function. The positive effect on functional mobility was further supported by the single study reporting the Timed Up and Go Test, which demonstrated significantly greater improvement in the BCI group compared to the control. The underlying mechanism likely involves the BCI system promoting the functional reorganization of cortical areas critical for postural control, by reinforcing the closed-loop integration of balance intention and successful execution (54–56). However, this result exhibited substantial heterogeneity. Through leave-one-out sensitivity analysis, we identified the study by Wan et al. (35) as the primary source of heterogeneity. Excluding this study significantly reduced heterogeneity (*I*^2^ = 0%). This might be due to differences in the “pedaling training” mode used in that study compared to the posture control and weight-bearing focused balance training modes in other studies (57), leading to inconsistent effects on balance improvement. After excluding this study, the beneficial effect of BCI on balance remained significant, indicating that the conclusion that BCI improves postural stability and dynamic balance by enhancing neural control over trunk and proximal lower limb muscles is reliable (58).

For the FMA-B and 6MWD, each outcome was reported by only a single study. No significant between-group differences were found for either measure. The current evidence remains insufficient to draw definitive conclusions regarding the effects of BCI on these specific aspects of balance and walking endurance.

### Effects of BCI on activities of daily living

The MBI is an important indicator for assessing patients' ability to perform ADL independently (59). This meta-analysis indicates that BCI training can improve patients' MBI scores, suggesting that BCI intervention helps enhance patients' independent living ability (48). However, this result exhibited extremely high heterogeneity. Our subgroup analyses provided a crucial insight into this issue, revealing that the cumulative treatment time was a significant moderator (*P* = 0.02). Interventions exceeding 500 min demonstrated a large, significant, and consistent effect on MBI, whereas shorter interventions yielded an uncertain and highly variable effect. This finding suggests that the observed overall heterogeneity largely stems from the dose-response relationship of BCI therapy. Other factors, such as differences in BCI systems, patient characteristics (e.g., time post-stroke, baseline disability), and varied rehabilitation foci, likely contribute further to the heterogeneity (45). Therefore, it can be concluded that while BCI shows promise in improving ADL, a sufficient cumulative dose is critical to achieving a reliable benefit, and the optimal parameters for different patient subgroups require further refinement.

### Subgroup analyses

We performed a set of subgroup analyses to identify the sources of heterogeneity and how stable the interventions' effects were. For the FMA-LE and BBS outcomes, parameters including treatment dosage patient age and crucially, the specific BCI paradigm, did not exhibit significant moderating effects. These non-significant findings firmly back up the primary results for these outcomes, suggesting that BCI is stably effective in improving motor impairment and balance across a range of clinical protocols age groups and technological implementations. This consistency supports the view that the core therapeutic element is the “intention-decoding–closed-loop feedback” process itself, which modulates corticospinal circuits irrespective of the peripheral actuator used. In contrast, for the MBI outcome, both cumulative treatment time and BCI paradigm significantly influenced results. The former indicates that the translation of BCI therapy into gains in daily living activities is highly dependent on receiving an adequate cumulative dose. These subgroup results sharpen the clinical application of BCI: its benefits for motor and balance function prove versatile and widely applicable, even across different stages of recovery, while its impact on ADL calls for a more intensive, structured approach to dosage. The latter indicates that the translation of neuromodulatory gains into real-world function depends critically on the specific nature of the peripheral interface. In this analysis, BCI+FES—which provides physiologically matched, somatotopic feedback-demonstrated a clear advantage over BCI+Active Cycling for improving activities of daily living. This refines the clinical application: while benefits for basic motor function are generalizable, optimizing ADL outcomes requires not only sufficient dosage but preferably pairing with a peripheral interface like FES that enables direct functional integration.

### Limitations

It is important to acknowledge the limitations of this study:

1. The number of studies included for some outcomes (such as MBI) was relatively limited, which may affect the stability of the pooled results (60).
2. Although all included studies were randomized controlled trials, most did not provide detailed descriptions of allocation concealment, potentially introducing risks of performance and detection bias (61).
3. Moderate to high heterogeneity was observed for the BBS and MBI outcomes. Although we attempted to explore its sources through sensitivity analysis, some heterogeneity remained unexplained, which might affect the precision of the conclusions (62).
4. All included studies were conducted in China, so the generalizability of the findings to other ethnicities and healthcare systems requires further validation (63). This concentration may limit the applicability of our results to settings with differing rehabilitation protocols, healthcare resources, patient demographics, or cultural contexts.
5. The included studies enrolled mixed stroke types without stratification, preventing subgroup analysis by etiology and limiting generalizability. Future studies should investigate BCI efficacy across different stroke subtypes.
6. The included studies varied in their reporting of stroke phase and seldom detailed concomitant pharmacotherapies. As both factors may influence treatment response and represent sources of heterogeneity, future trials should consistently report these clinical details to facilitate subgroup analysis and improve interpretability.

### Clinical and research implications

This study confirms BCI as an effective and reliable tool for improving lower limb motor and balance function in stroke patients. Most critically, the uncovered dose-response relationship for MBI (activities of daily living) offers a critical reference point for clinical practice, indicating that cumulative intervention plans of no fewer than 500 min prove critical to maximizing functional recovery. Future work should focus on precisely defining the minimum and optimal effective doses of BCI, and exploring patient subgroups most likely to benefit, as suggested by the current trends. Additionally, confirming these findings in large-scale, multicenter international trials is essential to establish their generalizability across diverse healthcare systems, rehabilitation protocols, and patient populations. Advancing the standardization and long-term efficacy evaluation of BCI technology are equally critical next steps. Furthermore, it is noteworthy that among the 13 included RCTs, only 4 were prospectively registered in the Chinese Clinical Trial Registry, while the remaining 9 did not provide registration information. Moving forward, prospective registration of clinical trials should be emphasized as a fundamental practice to enhance transparency, reduce reporting bias, and improve the overall methodological quality of future BCI research.

## Conclusion

Current evidence suggests that BCI-based training can effectively improve lower limb motor function, balance, and activities of daily living in stroke patients. The beneficial effect on balance was further corroborated by improvements in functional mobility. While its effect on improving motor function is particularly robust and consistent, findings for other specific measures require further investigation. As an emerging rehabilitation technology, BCI provides a new and effective strategy for stroke rehabilitation. Future research should include larger, high-quality, multi-center studies to verify its long-term efficacy, identify the optimal treatment protocols and target patient populations, and clarify its impact across a broader spectrum of functional outcomes.

## Data Availability

The original contributions presented in the study are included in the article/Supplementary material, further inquiries can be directed to the corresponding author.
