# Supplementary material for: Effects of brain-computer interface-based rehabilitation on lower limb function and activities of daily living after stroke: a systematic review and meta-analysis
Source: Front Neurol. 2026 Feb 25;17:1746958. doi: 10.3389/fneur.2026.1746958 (PMC12975493; doi:10.3389/fneur.2026.1746958)
Supplement: Supplementary file 1 [file Data_Sheet_1.pdf]

Supplement Figure 1.Forest Plot for the FMA-LE by Number of Treatment Sessions

	Experimental			Control				Mean Difference
Study or Subgroup	Mean	SD	Total	Mean	SD	Total	Weight	IV, Random, 95% CI
**4.1.1 < 20 sessions**								
Gao 2023	5.34	2.57	32	3.31	2.24	32	9.3%	2.03 [0.85, 3.21]
Huang JQ 2024	6.74	3.76	15	3.13	3.94	15	1.7%	3.61 [0.85, 6.37]
Li 2024	4.7	4.48	36	2.94	5.49	37	2.5%	1.76 [-0.54, 4.06]
Sun 2023	9	4.63	30	6	3.39	30	3.1%	3.00 [0.95, 5.05]
Zhai 2025	6.47	3.99	17	2.88	2.15	17	2.8%	3.59 [1.44, 5.74]
**Subtotal (95% CI)**			**130**			**131**	**19.3%**	**2.51 [1.70, 3.33]**
Heterogeneity: Tau² = 0.00; Chi² = 2.84, df = 4 (P = 0.58); I² = 0%								
Test for overall effect: Z = 6.02 (P < 0.00001)								
**4.1.2 ≥ 20 sessions**								
Fang 2018	12.95	2.94	20	9.68	2.26	20	4.9%	3.27 [1.64, 4.90]
Han 2025	8.9	0.85	14	6.17	0.7	14	39.0%	2.73 [2.15, 3.31]
Huang Y 2024	10	4.16	20	5.6	6.14	20	1.2%	4.40 [1.15, 7.65]
Jin 2025	8.76	4.6	24	4.67	6.58	25	1.3%	4.09 [0.92, 7.26]
Liu 2024	12.54	4.1	15	9.07	4.63	15	1.3%	3.47 [0.34, 6.60]
Luo 2025	5.53	1.35	32	2.91	1.45	32	27.5%	2.62 [1.93, 3.31]
Tang 2024	6.05	3.48	20	3.31	4.59	20	2.0%	2.74 [0.22, 5.26]
Wan 2025	4.36	2.68	14	3.63	2.85	16	3.3%	0.73 [-1.25, 2.71]
**Subtotal (95% CI)**			**159**			**162**	**80.7%**	**2.70 [2.30, 3.10]**
Heterogeneity: Tau² = 0.00; Chi² = 6.36, df = 7 (P = 0.50); I² = 0%								
Test for overall effect: Z = 13.21 (P < 0.00001)								
**Total (95% CI)**			**289**			**293**	**100.0%**	**2.67 [2.31, 3.03]**
Heterogeneity: Tau² = 0.00; Chi² = 9.36, df = 12 (P = 0.67); I² = 0%								
Test for overall effect: Z = 14.51 (P < 0.00001)								
Test for subgroup differences: Chi² = 0.16, df = 1 (P = 0.69), I² = 0%								

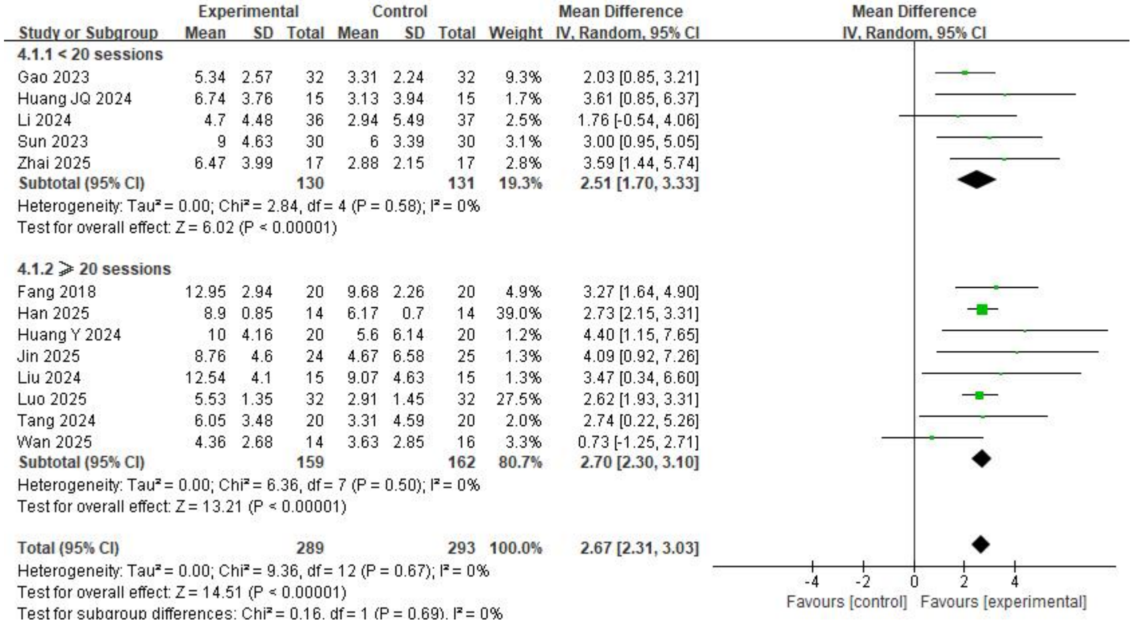

Supplement Figure 2.Forest Plot for the FMA-LE by Intervention Duration

	Experimental			Control				Mean Difference
Study or Subgroup	Mean	SD	Total	Mean	SD	Total	Weight	IV, Random, 95% CI
**5.1.1 Treatment: < 4 Weeks**								
Gao 2023	5.34	2.57	32	3.31	2.24	32	9.3%	2.03 [0.85, 3.21]
Huang JQ 2024	6.74	3.76	15	3.13	3.94	15	1.7%	3.61 [0.85, 6.37]
Li 2024	4.7	4.48	36	2.94	5.49	37	2.5%	1.76 [-0.54, 4.06]
Luo 2025	5.53	1.35	32	2.91	1.45	32	27.5%	2.62 [1.93, 3.31]
Sun 2023	9	4.63	30	6	3.39	30	3.1%	3.00 [0.95, 5.05]
Zhai 2025	6.47	3.99	17	2.88	2.15	17	2.8%	3.59 [1.44, 5.74]
**Subtotal (95% CI)**			**162**			**163**	**46.9%**	**2.58 [2.05, 3.10]**
Heterogeneity: Tau² = 0.00; Chi² = 2.88, df = 5 (P = 0.72); I² = 0%								
Test for overall effect: Z = 9.60 (P < 0.00001)								
**5.1.2 Treatment: ⩾ 4 Weeks**								
Fang 2018	12.95	2.94	20	9.68	2.26	20	4.9%	3.27 [1.64, 4.90]
Han 2025	8.9	0.85	14	6.17	0.7	14	39.0%	2.73 [2.15, 3.31]
Huang Y 2024	10	4.16	20	5.6	6.14	20	1.2%	4.40 [1.15, 7.65]
Jin 2025	8.76	4.6	24	4.67	6.58	25	1.3%	4.09 [0.92, 7.26]
Liu 2024	12.54	4.1	15	9.07	4.63	15	1.3%	3.47 [0.34, 6.60]
Tang 2024	6.05	3.48	20	3.31	4.59	20	2.0%	2.74 [0.22, 5.26]
Wan 2025	4.36	2.68	14	3.63	2.85	16	3.3%	0.73 [-1.25, 2.71]
**Subtotal (95% CI)**			**127**			**130**	**53.1%**	**2.75 [2.19, 3.32]**
Heterogeneity: Tau² = 0.04; Chi² = 6.28, df = 6 (P = 0.39); I² = 4%								
Test for overall effect: Z = 9.52 (P < 0.00001)								
**Total (95% CI)**			**289**			**293**	**100.0%**	**2.67 [2.31, 3.03]**
Heterogeneity: Tau² = 0.00; Chi² = 9.36, df = 12 (P = 0.67); I² = 0%								
Test for overall effect: Z = 14.51 (P < 0.00001)								
Test for subgroup differences: Chi² = 0.20, df = 1 (P = 0.66), I² = 0%								

Mean Difference
IV, Random, 95% CI

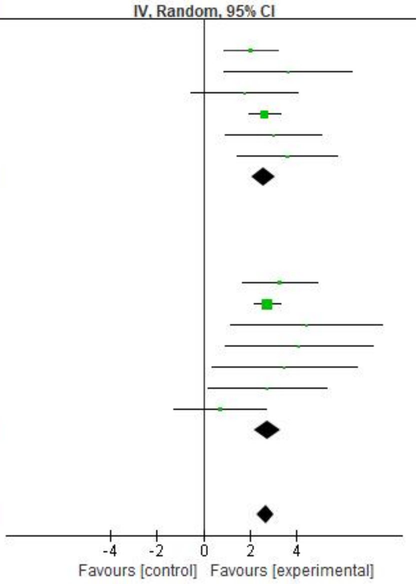

Supplement Figure 3.Forest Plot for the FMA-LE by Cumulative Treatment Time

	Experimental			Control				Mean Difference
Study or Subgroup	Mean	SD	Total	Mean	SD	Total	Weight	IV, Random, 95% CI
**6.1.1 Cumulative treatment time < 500 min**								
Gao 2023	5.34	2.57	32	3.31	2.24	32	9.6%	2.03 [0.85, 3.21]
Han 2025	8.9	0.85	14	6.17	0.7	14	40.2%	2.73 [2.15, 3.31]
Huang JQ 2024	6.74	3.76	15	3.13	3.94	15	1.8%	3.61 [0.85, 6.37]
Huang Y 2024	10	4.16	20	5.6	6.14	20	1.3%	4.40 [1.15, 7.65]
Li 2024	4.7	4.48	36	2.94	5.49	37	2.5%	1.76 [-0.54, 4.06]
Tang 2024	6.05	3.48	20	3.31	4.59	20	2.1%	2.74 [0.22, 5.26]
Wan 2025	4.36	2.68	14	3.63	2.85	16	3.4%	0.73 [-1.25, 2.71]
Zhai 2025	6.47	3.99	17	2.88	2.15	17	2.9%	3.59 [1.44, 5.74]
**Subtotal (95% CI)**			**168**			**171**	**63.8%**	**2.54 [2.01, 3.08]**
Heterogeneity: Tau² = 0.05; Chi² = 7.54, df = 7 (P = 0.38); I² = 7%								
Test for overall effect: Z = 9.36 (P < 0.00001)								
**6.1.2 Cumulative treatment time ⩾ 500 min**								
Fang 2018	12.95	2.94	20	9.68	2.26	20	5.1%	3.27 [1.64, 4.90]
Jin 2025	8.76	4.6	24	4.67	6.58	25	1.3%	4.09 [0.92, 7.26]
Liu 2024	12.54	4.1	15	9.07	4.63	15	1.4%	3.47 [0.34, 6.60]
Luo 2025	5.53	1.35	32	2.91	1.45	32	28.4%	2.62 [1.93, 3.31]
**Subtotal (95% CI)**			**91**			**92**	**36.2%**	**2.80 [2.19, 3.41]**
Heterogeneity: Tau² = 0.00; Chi² = 1.40, df = 3 (P = 0.71); I² = 0%								
Test for overall effect: Z = 9.01 (P < 0.00001)								
**Total (95% CI)**			**259**			**263**	**100.0%**	**2.66 [2.29, 3.02]**
Heterogeneity: Tau² = 0.00; Chi² = 9.26, df = 11 (P = 0.60); I² = 0%								
Test for overall effect: Z = 14.23 (P < 0.00001)								
Test for subgroup differences: Chi² = 0.38, df = 1 (P = 0.54), I² = 0%								

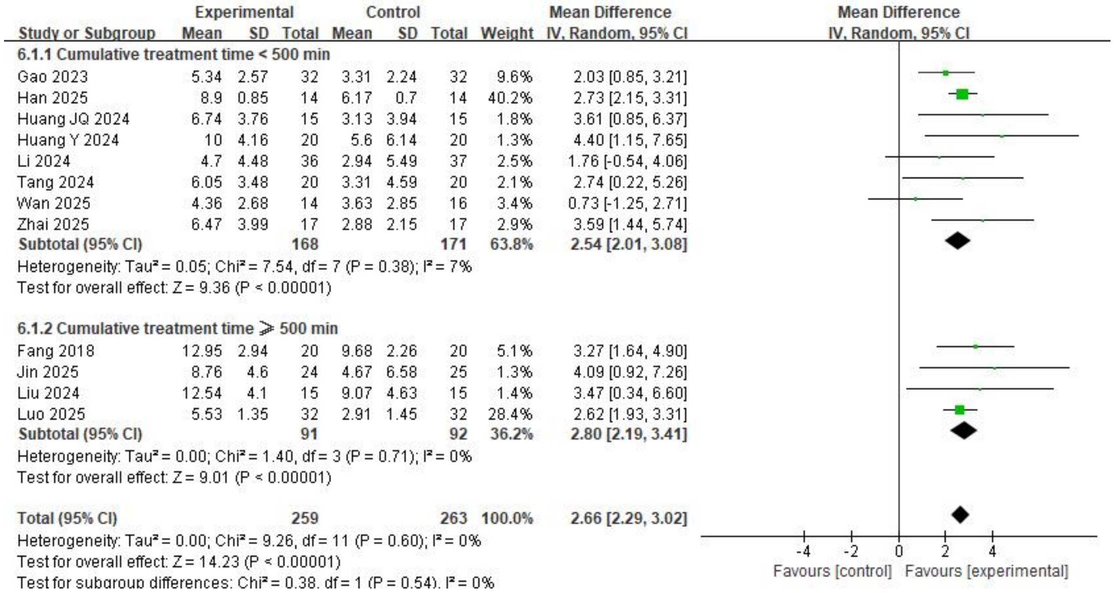

Supplement Figure 4.Forest Plot for the FMA-LE by Mean Patient Age

Study or Subgroup	Experimental Mean	Experimental SD	Experimental Total	Control Mean	Control SD	Control Total	Weight	Mean Difference IV, Random, 95% CI
**7.1.1 Mean age ⩽55 years**								
Gao 2023	5.34	2.57	32	3.31	2.24	32	12.8%	2.03 [0.85, 3.21]
Han 2025	8.9	0.85	14	6.17	0.7	14	53.8%	2.73 [2.15, 3.31]
Jin 2025	8.76	4.6	24	4.67	6.58	25	1.8%	4.09 [0.92, 7.26]
Li 2024	4.7	4.48	36	2.94	5.49	37	3.4%	1.76 [-0.54, 4.06]
Liu 2024	12.54	4.1	15	9.07	4.63	15	1.8%	3.47 [0.34, 6.60]
Sun 2023	9	4.63	30	6	3.39	30	4.2%	3.00 [0.95, 5.05]
**Subtotal (95% CI)**			**151**			**153**	**77.9%**	**2.64 [2.16, 3.12]**
Heterogeneity: Tau² = 0.00; Chi² = 2.87, df = 5 (P = 0.72); I² = 0%								
Test for overall effect: Z = 10.78 (P < 0.00001)								
**7.1.2 Mean age >55 years**								
Fang 2018	12.95	2.94	20	9.68	2.26	20	6.8%	3.27 [1.64, 4.90]
Huang JQ 2024	6.74	3.76	15	3.13	3.94	15	2.4%	3.61 [0.85, 6.37]
Huang Y 2024	10	4.16	20	5.6	6.14	20	1.7%	4.40 [1.15, 7.65]
Tang 2024	6.05	3.48	20	3.31	4.59	20	2.8%	2.74 [0.22, 5.26]
Wan 2025	4.36	2.68	14	3.63	2.85	16	4.6%	0.73 [-1.25, 2.71]
Zhai 2025	6.47	3.99	17	2.88	2.15	17	3.9%	3.59 [1.44, 5.74]
**Subtotal (95% CI)**			**106**			**108**	**22.1%**	**2.88 [1.85, 3.91]**
Heterogeneity: Tau² = 0.34; Chi² = 6.29, df = 5 (P = 0.28); I² = 20%								
Test for overall effect: Z = 5.47 (P < 0.00001)								
**Total (95% CI)**			**257**			**261**	**100.0%**	**2.68 [2.26, 3.11]**
Heterogeneity: Tau² = 0.00; Chi² = 9.34, df = 11 (P = 0.59); I² = 0%								
Test for overall effect: Z = 12.43 (P < 0.00001)								
Test for subgroup differences: Chi² = 0.18, df = 1 (P = 0.68), I² = 0%								

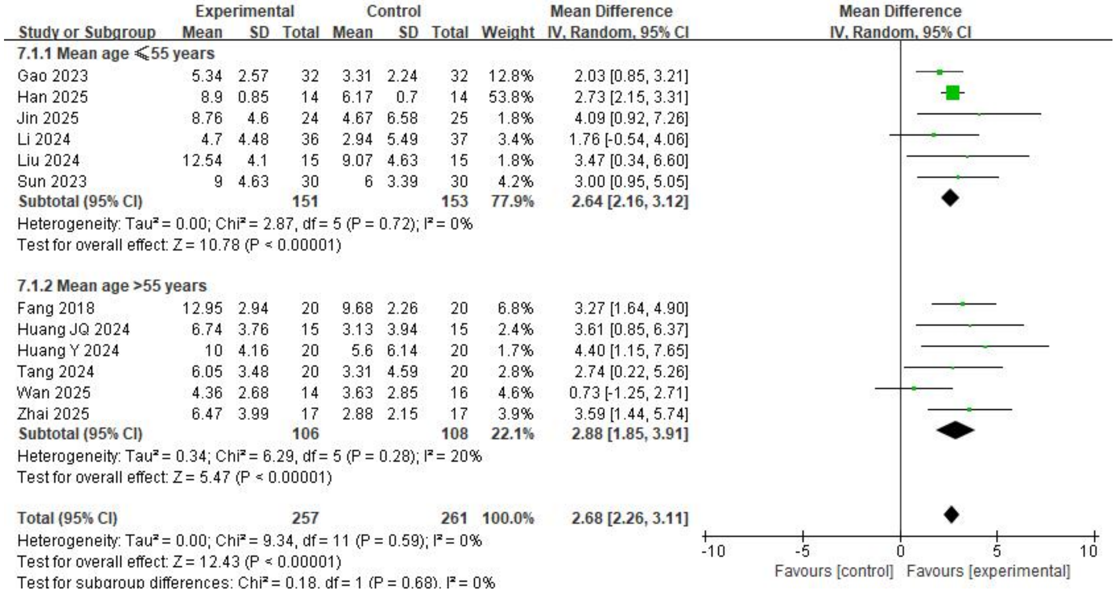

Supplement Figure 5.Forest Plot for the FMA-LE by disease course

Study or Subgroup	Control Mean	Control SD	Control Total	Experimental Mean	Experimental SD	Experimental Total	Weight	Mean Difference IV, Fixed, 95% CI
**15.1.1 ≤ 90 days**								
Fang 2018	12.95	2.94	20	9.68	2.26	20	9.9%	3.27 [1.64, 4.90]
Liu 2024	12.54	4.1	15	9.07	4.63	15	2.7%	3.47 [0.34, 6.60]
Luo 2025	5.53	1.35	32	2.91	1.45	32	55.5%	2.62 [1.93, 3.31]
Tang 2024	6.05	3.48	20	3.31	4.59	20	4.1%	2.74 [0.22, 5.26]
Zhai 2025	6.47	3.99	17	2.88	2.15	17	5.6%	3.59 [1.44, 5.74]
**Subtotal (95% CI)**			**104**			**104**	**77.8%**	**2.81 [2.23, 3.39]**
Heterogeneity: Chi² = 1.28, df = 4 (P = 0.86); I² = 0%								
Test for overall effect: Z = 9.49 (P < 0.00001)								
**15.1.2 > 90 days**								
Gao 2023	5.34	2.57	32	3.31	2.24	32	18.7%	2.03 [0.85, 3.21]
Huang JQ 2024	6.74	3.76	15	3.13	3.94	15	3.4%	3.61 [0.85, 6.37]
**Subtotal (95% CI)**			**47**			**47**	**22.2%**	**2.28 [1.19, 3.36]**
Heterogeneity: Chi² = 1.07, df = 1 (P = 0.30); I² = 6%								
Test for overall effect: Z = 4.11 (P < 0.0001)								
**Total (95% CI)**			**151**			**151**	**100.0%**	**2.69 [2.18, 3.20]**
Heterogeneity: Chi² = 3.07, df = 6 (P = 0.80); I² = 0%								
Test for overall effect: Z = 10.31 (P < 0.00001)								
Test for subgroup differences: Chi² = 0.72, df = 1 (P = 0.40), I² = 0%								

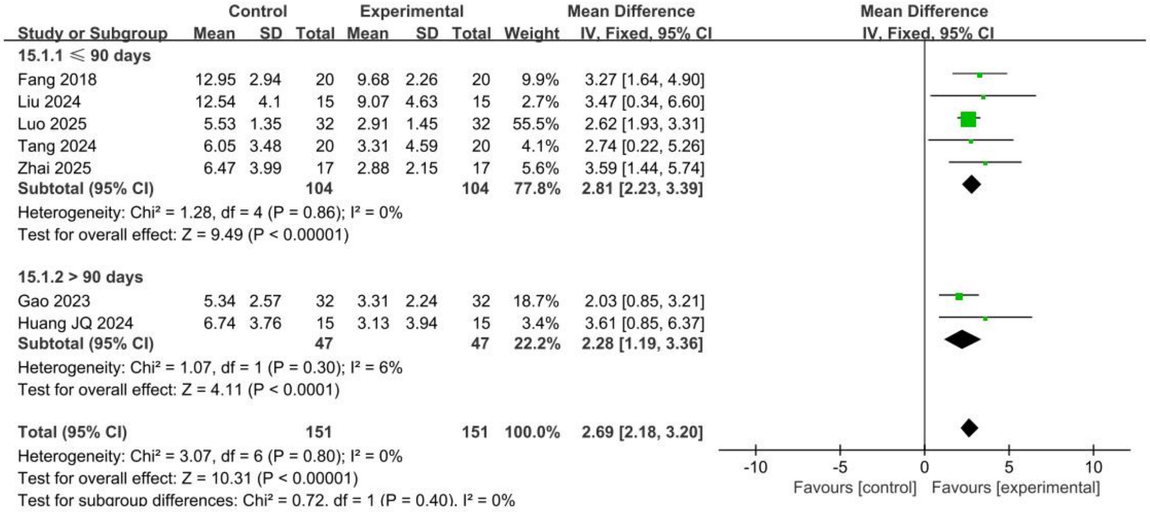

Supplement Figure 6.Forest Plot for the FMA-LE by BCI paradigm

Study or Subgroup	Experimental Mean	Experimental SD	Experimental Total	Control Mean	Control SD	Control Total	Weight	Mean Difference IV, Fixed, 95% CI
**15.2.1 BCI+Pedaling**								
Fang 2018	12.95	2.94	20	9.68	2.26	20	4.9%	3.27 [1.64, 4.90]
Gao 2023	5.34	2.57	32	3.31	2.24	32	9.3%	2.03 [0.85, 3.21]
Han 2025	8.9	0.85	14	6.17	0.7	14	39.0%	2.73 [2.15, 3.31]
Huang JQ 2024	6.74	3.76	15	3.13	3.94	15	1.7%	3.61 [0.85, 6.37]
Huang Y 2024	10	4.16	20	5.6	6.14	20	1.2%	4.40 [1.15, 7.65]
Jin 2025	8.76	4.6	24	4.67	6.58	25	1.3%	4.09 [0.92, 7.26]
Li 2024	4.7	4.48	36	2.94	5.49	37	2.5%	1.76 [-0.54, 4.06]
Tang 2024	6.05	3.48	20	3.31	4.59	20	2.0%	2.74 [0.22, 5.26]
Zhai 2025	6.47	3.99	17	2.88	2.15	17	2.8%	3.59 [1.44, 5.74]
**Subtotal (95% CI)**			**198**			**200**	**64.7%**	**2.75 [2.31, 3.20]**
Heterogeneity: Chi² = 5.17, df = 8 (P = 0.74); I² = 0%								
Test for overall effect: Z = 12.05 (P < 0.00001)								
**15.2.2 BCI+FES**								
Liu 2024	12.54	4.1	15	9.07	4.63	15	1.3%	3.47 [0.34, 6.60]
Luo 2025	5.53	1.35	32	2.91	1.45	32	27.5%	2.62 [1.93, 3.31]
Sun 2023	9	4.63	30	6	3.39	30	3.1%	3.00 [0.95, 5.05]
Wan 2025	4.36	2.68	14	3.63	2.85	16	3.3%	0.73 [-1.25, 2.71]
**Subtotal (95% CI)**			**91**			**93**	**35.3%**	**2.51 [1.90, 3.11]**
Heterogeneity: Chi² = 3.78, df = 3 (P = 0.29); I² = 21%								
Test for overall effect: Z = 8.10 (P < 0.00001)								
**Total (95% CI)**			**289**			**293**	**100.0%**	**2.67 [2.31, 3.03]**
Heterogeneity: Chi² = 9.36, df = 12 (P = 0.67); I² = 0%								
Test for overall effect: Z = 14.51 (P < 0.00001)								
Test for subgroup differences: Chi² = 0.41, df = 1 (P = 0.52), I² = 0%								

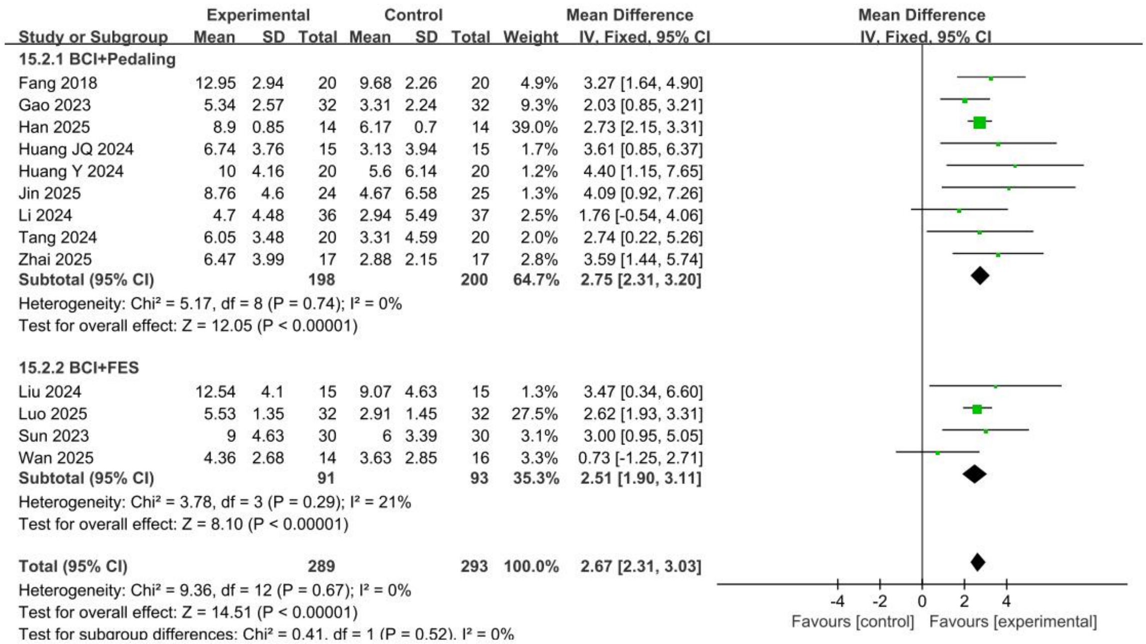

Supplement Figure 7.Forest Plot for the BBS by Number of Treatment Sessions

	Experimental			Control				Mean Difference
Study or Subgroup	Mean	SD	Total	Mean	SD	Total	Weight	IV, Random, 95% CI
**8.1.1 <20 sessions**								
Sun 2023	17.03	9.56	30	3	25.22	30	10.3%	14.03 [4.38, 23.68]
Zhai 2025	12	21.95	17	5.9	10.52	17	8.1%	6.10 [-5.47, 17.67]
**Subtotal (95% CI)**			**47**			**47**	**18.4%**	**10.73 [3.07, 18.39]**
Heterogeneity: Tau² = 1.89; Chi² = 1.06, df = 1 (P = 0.30); I² = 6%								
Test for overall effect: Z = 2.75 (P = 0.006)								
**8.1.2 >20 sessions**								
Huang Y 2024	16.6	10.32	20	10.07	7.93	20	17.6%	6.53 [0.83, 12.23]
Liu 2024	17.2	6.87	15	7.68	7.78	15	18.7%	9.52 [4.27, 14.77]
Tang 2024	11.85	7.01	20	3.37	9.32	20	19.0%	8.48 [3.37, 13.59]
Wan 2025	5.5	3.11	14	3.38	2.34	16	26.3%	2.12 [0.13, 4.11]
**Subtotal (95% CI)**			**69**			**71**	**81.6%**	**6.22 [2.03, 10.40]**
Heterogeneity: Tau² = 12.88; Chi² = 11.36, df = 3 (P = 0.010); I² = 74%								
Test for overall effect: Z = 2.91 (P = 0.004)								
**Total (95% CI)**			**116**			**118**	**100.0%**	**7.04 [3.14, 10.94]**
Heterogeneity: Tau² = 14.01; Chi² = 15.55, df = 5 (P = 0.008); I² = 68%								
Test for overall effect: Z = 3.54 (P = 0.0004)								
Test for subgroup differences: Chi² = 1.03, df = 1 (P = 0.31), I² = 2.8%								

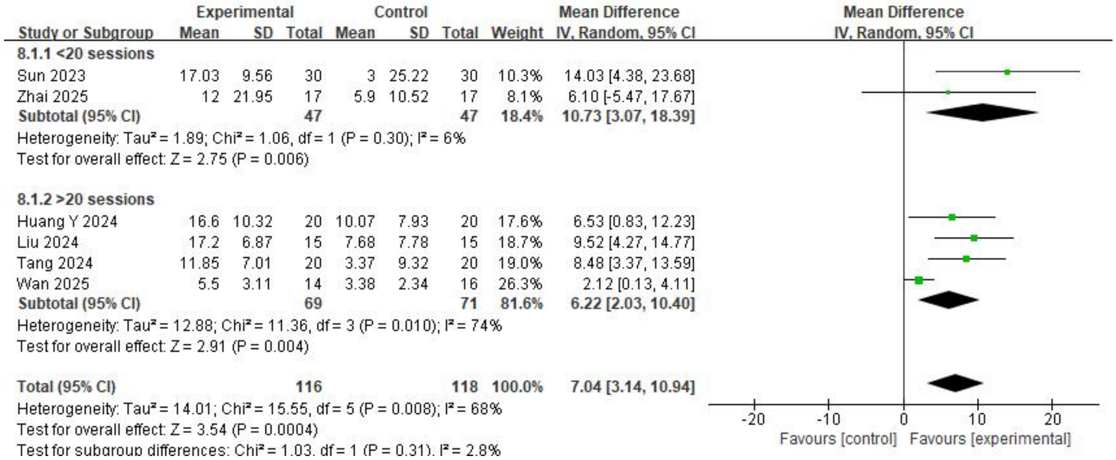

Supplement Figure 8.Forest Plot for the BBS by Intervention Duration

	Experimental			Control				Mean Difference
Study or Subgroup	Mean	SD	Total	Mean	SD	Total	Weight	IV, Random, 95% CI
**9.1.1 <4 weeks**								
Sun 2023	17.03	9.56	30	3	25.22	30	10.3%	14.03 [4.38, 23.68]
Zhai 2025	12	21.95	17	5.9	10.52	17	8.1%	6.10 [-5.47, 17.67]
**Subtotal (95% CI)**			**47**			**47**	**18.4%**	**10.73 [3.07, 18.39]**
Heterogeneity: Tau² = 1.89; Chi² = 1.06, df = 1 (P = 0.30); I² = 6%								
Test for overall effect: Z = 2.75 (P = 0.006)								
**9.1.2 ⩾4 weeks**								
Huang Y 2024	16.6	10.32	20	10.07	7.93	20	17.6%	6.53 [0.83, 12.23]
Liu 2024	17.2	6.87	15	7.68	7.78	15	18.7%	9.52 [4.27, 14.77]
Tang 2024	11.85	7.01	20	3.37	9.32	20	19.0%	8.48 [3.37, 13.59]
Wan 2025	5.5	3.11	14	3.38	2.34	16	26.3%	2.12 [0.13, 4.11]
**Subtotal (95% CI)**			**69**			**71**	**81.6%**	**6.22 [2.03, 10.40]**
Heterogeneity: Tau² = 12.88; Chi² = 11.36, df = 3 (P = 0.010); I² = 74%								
Test for overall effect: Z = 2.91 (P = 0.004)								
**Total (95% CI)**			**116**			**118**	**100.0%**	**7.04 [3.14, 10.94]**
Heterogeneity: Tau² = 14.01; Chi² = 15.55, df = 5 (P = 0.008); I² = 68%								
Test for overall effect: Z = 3.54 (P = 0.0004)								
Test for subgroup differences: Chi² = 1.03, df = 1 (P = 0.31), I² = 2.8%								

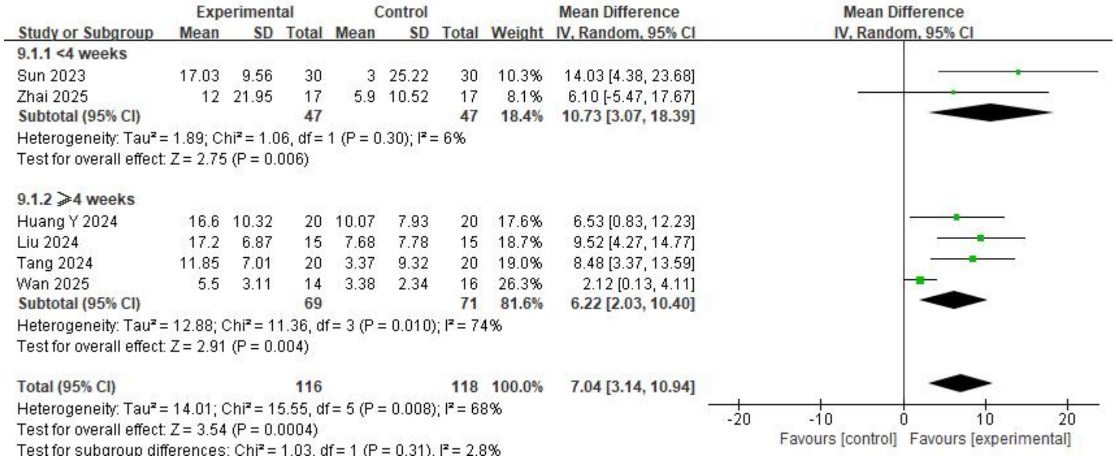

Supplement Figure 9.Forest Plot for the BBS by Cumulative Treatment Time

	Experimental			Control				Mean Difference
Study or Subgroup	Mean	SD	Total	Mean	SD	Total	Weight	IV, Random, 95% CI
**10.1.1 <500 minutes**								
Huang Y 2024	16.6	10.32	20	10.07	7.93	20	19.2%	6.53 [0.83, 12.23]
Tang 2024	11.85	7.01	20	3.37	9.32	20	21.0%	8.48 [3.37, 13.59]
Wan 2025	5.5	3.11	14	3.38	2.34	16	31.2%	2.12 [0.13, 4.11]
Zhai 2025	12	21.95	17	5.9	10.52	17	8.1%	6.10 [-5.47, 17.67]
**Subtotal (95% CI)**			**71**			**73**	**79.5%**	**5.11 [1.34, 8.88]**
Heterogeneity: Tau² = 7.64; Chi² = 6.75, df = 3 (P = 0.08); I² = 56%								
Test for overall effect: Z = 2.66 (P = 0.008)								
**10.1.2 ⩾500 minutes**								
Liu 2024	17.2	6.87	15	7.68	7.78	15	20.5%	9.52 [4.27, 14.77]
**Subtotal (95% CI)**			**15**			**15**	**20.5%**	**9.52 [4.27, 14.77]**
Heterogeneity: Not applicable								
Test for overall effect: Z = 3.55 (P = 0.0004)								
**Total (95% CI)**			**86**			**88**	**100.0%**	**6.14 [2.37, 9.91]**
Heterogeneity: Tau² = 10.83; Chi² = 11.50, df = 4 (P = 0.02); I² = 65%								
Test for overall effect: Z = 3.19 (P = 0.001)								
Test for subgroup differences: Chi² = 1.79, df = 1 (P = 0.18), I² = 44.0%								

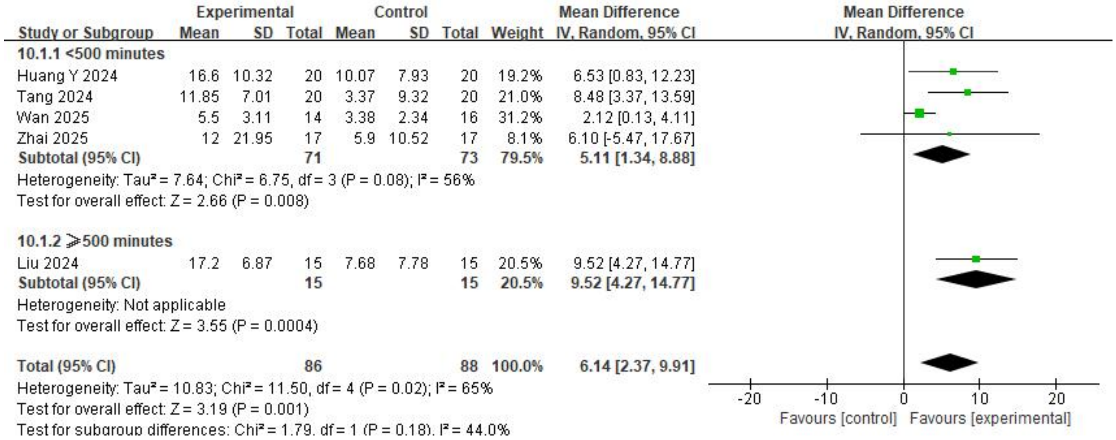

Supplement Figure 10.Forest Plot for the BBS by Mean Patient Age

	Experimental			Control				Mean Difference
Study or Subgroup	Mean	SD	Total	Mean	SD	Total	Weight	IV, Random, 95% CI
**11.1.1 ⩽55 years**								
Liu 2024	17.2	6.87	15	7.68	7.78	15	18.7%	9.52 [4.27, 14.77]
Sun 2023	17.03	9.56	30	3	25.22	30	10.3%	14.03 [4.38, 23.68]
**Subtotal (95% CI)**			**45**			**45**	**29.0%**	**10.55 [5.94, 15.16]**
Heterogeneity: $Tau^2 = 0.00$; $Chi^2 = 0.65$, df = 1 (P = 0.42); $I^2 = 0\%$								
Test for overall effect: Z = 4.48 (P < 0.00001)								
**11.1.2 >55 years**								
Huang Y 2024	16.6	10.32	20	10.07	7.93	20	17.6%	6.53 [0.83, 12.23]
Tang 2024	11.85	7.01	20	3.37	9.32	20	19.0%	8.48 [3.37, 13.59]
Wan 2025	5.5	3.11	14	3.38	2.34	16	26.3%	2.12 [0.13, 4.11]
Zhai 2025	12	21.95	17	5.9	10.52	17	8.1%	6.10 [-5.47, 17.67]
**Subtotal (95% CI)**			**71**			**73**	**71.0%**	**5.11 [1.34, 8.88]**
Heterogeneity: $Tau^2 = 7.64$; $Chi^2 = 6.75$, df = 3 (P = 0.08); $I^2 = 56\%$								
Test for overall effect: Z = 2.66 (P = 0.008)								
**Total (95% CI)**			**116**			**118**	**100.0%**	**7.04 [3.14, 10.94]**
Heterogeneity: $Tau^2 = 14.01$; $Chi^2 = 15.55$, df = 5 (P = 0.008); $I^2 = 68\%$								
Test for overall effect: Z = 3.54 (P = 0.0004)								
Test for subgroup differences: $Chi^2 = 3.20$, df = 1 (P = 0.07), $I^2 = 68.8\%$								

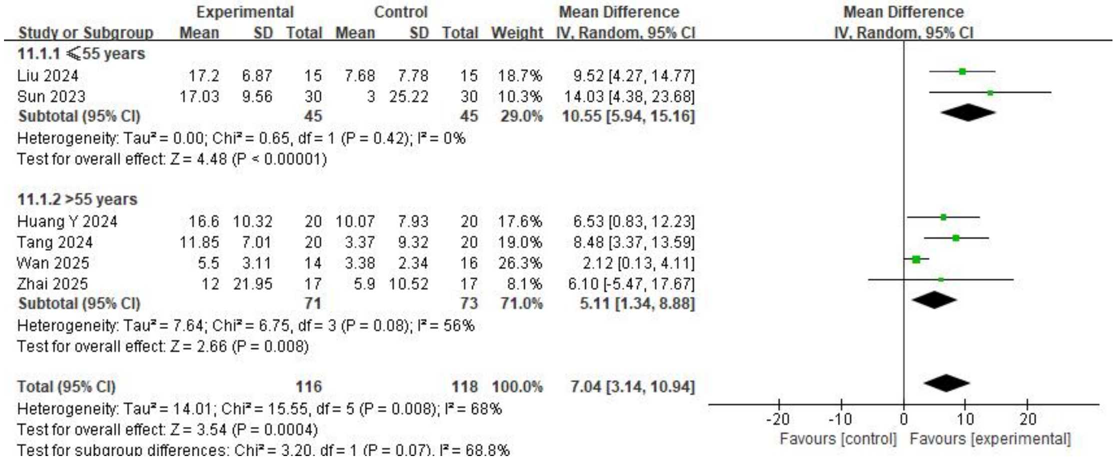

Supplement Figure 11.Forest Plot for the BBS by BCI paradigm

Study or Subgroup	Experimental Mean	Experimental SD	Experimental Total	Control Mean	Control SD	Control Total	Weight	Mean Difference IV, Random, 95% CI
**16.1.1 BCI+Pedaling**								
Huang Y 2024	16.6	10.32	20	10.07	7.93	20	17.6%	6.53 [0.83, 12.23]
Tang 2024	11.85	7.01	20	3.37	9.32	20	19.0%	8.48 [3.37, 13.59]
Zhai 2025	12	21.95	17	5.9	10.52	17	8.1%	6.10 [-5.47, 17.67]
**Subtotal (95% CI)**			**57**			**57**	**44.7%**	**7.46 [3.85, 11.08]**
Heterogeneity: Tau² = 0.00; Chi² = 0.31, df = 2 (P = 0.86); I² = 0%								
Test for overall effect: Z = 4.05 (P < 0.0001)								
**16.1.2 BCI+FES**								
Liu 2024	17.2	6.87	15	7.68	7.78	15	18.7%	9.52 [4.27, 14.77]
Sun 2023	17.03	9.56	30	3	25.22	30	10.3%	14.03 [4.38, 23.68]
Wan 2025	5.5	3.11	14	3.38	2.34	16	26.3%	2.12 [0.13, 4.11]
**Subtotal (95% CI)**			**59**			**61**	**55.3%**	**7.52 [0.47, 14.57]**
Heterogeneity: Tau² = 30.10; Chi² = 11.46, df = 2 (P = 0.003); I² = 83%								
Test for overall effect: Z = 2.09 (P = 0.04)								
**Total (95% CI)**			**116**			**118**	**100.0%**	**7.04 [3.14, 10.94]**

Heterogeneity: Tau² = 14.01; Chi² = 15.55, df = 5 (P = 0.008); I² = 68%
Test for overall effect: Z = 3.54 (P = 0.0004)
Test for subgroup differences: Chi² = 0.00, df = 1 (P = 0.99), I² = 0%

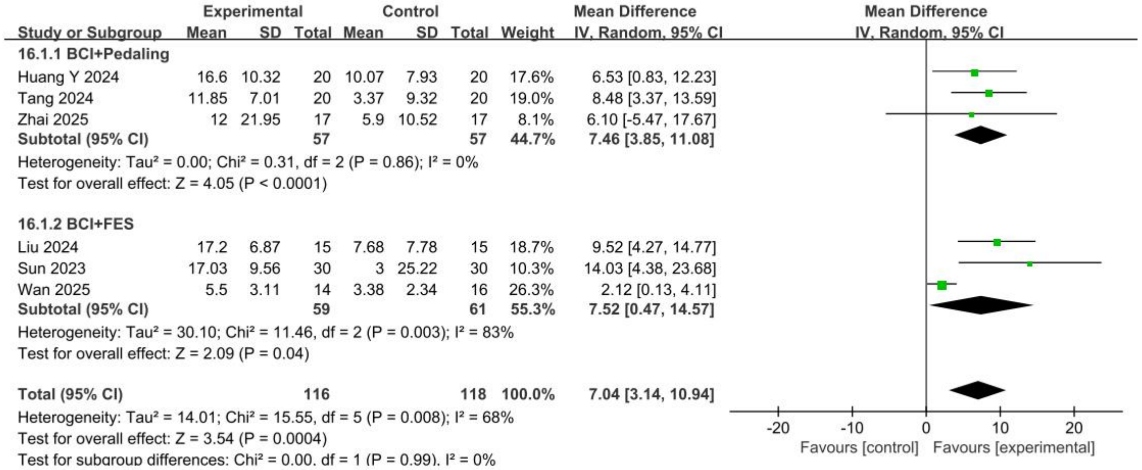

Supplement Figure 12.Forest Plot for the MBI by Number of Treatment Sessions

	Experimental			Control				Mean Difference
Study or Subgroup	Mean	SD	Total	Mean	SD	Total	Weight	IV, Random, 95% CI
**12.1.1 <20 sessions**								
Gao 2023	6.72	3.27	32	5.78	3.14	32	23.4%	0.94 [-0.63, 2.51]
Sun 2023	33.7	11.09	30	26.53	15.91	30	16.3%	7.17 [0.23, 14.11]
**Subtotal (95% CI)**			**62**			**62**	**39.8%**	**3.10 [-2.71, 8.91]**
Heterogeneity: Tau² = 12.82; Chi² = 2.94, df = 1 (P = 0.09); I² = 66%								
Test for overall effect: Z = 1.05 (P = 0.30)								
**12.1.2 ≥20 sessions**								
Han 2025	24.13	2.84	14	17.38	2.21	14	23.2%	6.75 [4.86, 8.64]
Liu 2024	26.33	11.51	15	17.67	13.38	15	13.5%	8.66 [-0.27, 17.59]
Luo 2025	19	3.23	32	8	2.22	32	23.5%	11.00 [9.64, 12.36]
**Subtotal (95% CI)**			**61**			**61**	**60.2%**	**8.90 [5.24, 12.56]**
Heterogeneity: Tau² = 7.28; Chi² = 12.89, df = 2 (P = 0.002); I² = 84%								
Test for overall effect: Z = 4.76 (P < 0.00001)								
**Total (95% CI)**			**123**			**123**	**100.0%**	**6.72 [1.74, 11.69]**
Heterogeneity: Tau² = 26.89; Chi² = 90.37, df = 4 (P < 0.00001); I² = 96%								
Test for overall effect: Z = 2.65 (P = 0.008)								
Test for subgroup differences: Chi² = 2.74, df = 1 (P = 0.10), I² = 63.5%								

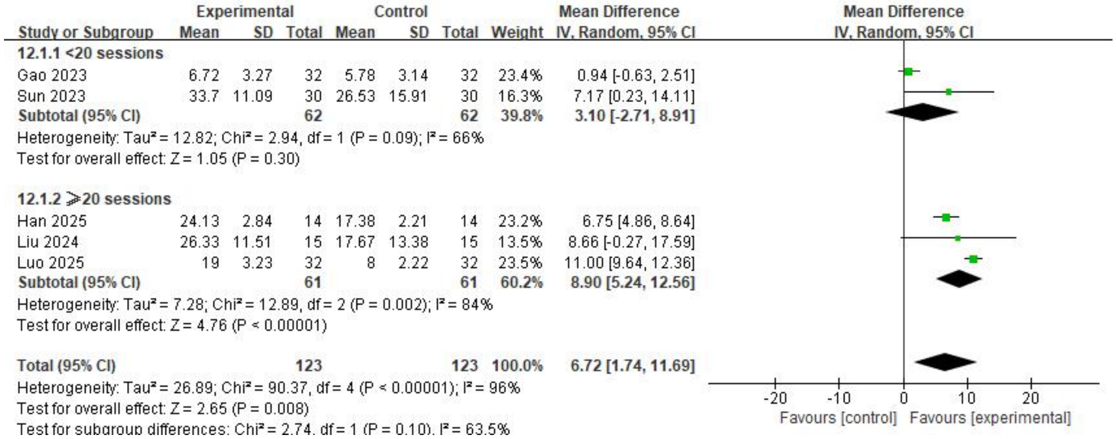

Supplement Figure 13.Forest Plot for the MBI by Intervention Duration

Study or Subgroup	Experimental Mean	Experimental SD	Experimental Total	Control Mean	Control SD	Control Total	Weight	Mean Difference IV, Random, 95% CI
**13.1.1 <4 weeks**								
Gao 2023	6.72	3.27	32	5.78	3.14	32	23.4%	0.94 [-0.63, 2.51]
Luo 2025	19	3.23	32	8	2.22	32	23.5%	11.00 [9.64, 12.36]
Sun 2023	33.7	11.09	30	26.53	15.91	30	16.3%	7.17 [0.23, 14.11]
**Subtotal (95% CI)**			**94**			**94**	**63.3%**	**6.32 [-1.71, 14.35]**
Heterogeneity: Tau² = 46.42; Chi² = 90.19, df = 2 (P < 0.00001); I² = 98%								
Test for overall effect: Z = 1.54 (P = 0.12)								
**13.1.2 ≥4 weeks**								
Han 2025	24.13	2.84	14	17.38	2.21	14	23.2%	6.75 [4.86, 8.64]
Liu 2024	26.33	11.51	15	17.67	13.38	15	13.5%	8.66 [-0.27, 17.59]
**Subtotal (95% CI)**			**29**			**29**	**36.7%**	**6.83 [4.99, 8.68]**
Heterogeneity: Tau² = 0.00; Chi² = 0.17, df = 1 (P = 0.68); I² = 0%								
Test for overall effect: Z = 7.26 (P < 0.00001)								
**Total (95% CI)**			**123**			**123**	**100.0%**	**6.72 [1.74, 11.69]**
Heterogeneity: Tau² = 26.89; Chi² = 90.37, df = 4 (P < 0.00001); I² = 96%								
Test for overall effect: Z = 2.65 (P = 0.008)								
Test for subgroup differences: Chi² = 0.01, df = 1 (P = 0.90), I² = 0%								

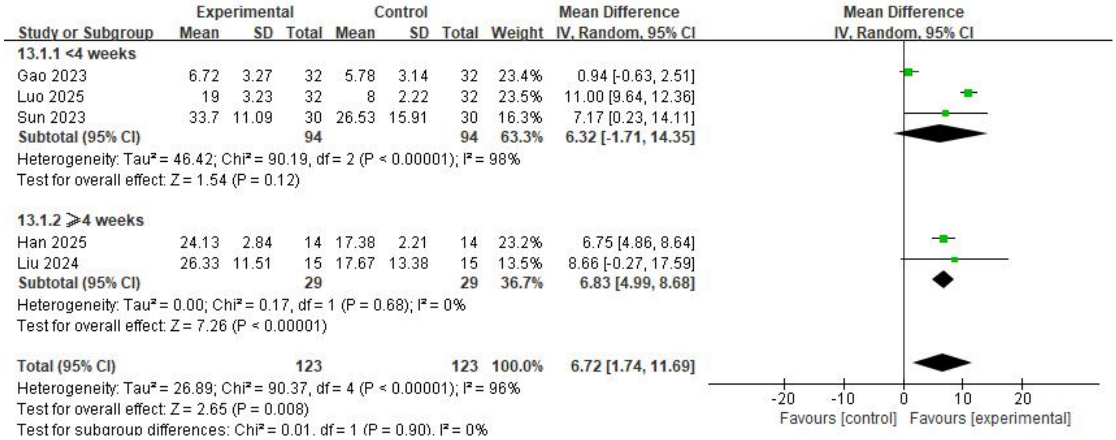

Supplement Figure 14.Forest Plot for the MBI by Cumulative Treatment Time

	Experimental			Control				Mean Difference
Study or Subgroup	Mean	SD	Total	Mean	SD	Total	Weight	IV, Random, 95% CI
**14.1.1 <500 minutes**								
Gao 2023	6.72	3.27	32	5.78	3.14	32	27.9%	0.94 [-0.63, 2.51]
Han 2025	24.13	2.84	14	17.38	2.21	14	27.6%	6.75 [4.86, 8.64]
**Subtotal (95% CI)**			**46**			**46**	**55.5%**	**3.82 [-1.87, 9.51]**
Heterogeneity: Tau² = 16.09; Chi² = 21.54, df = 1 (P < 0.00001); I² = 95%								
Test for overall effect: Z = 1.32 (P = 0.19)								
**14.1.2 ≥500 minutes**								
Liu 2024	26.33	11.51	15	17.67	13.38	15	16.4%	8.66 [-0.27, 17.59]
Luo 2025	19	3.23	32	8	2.22	32	28.1%	11.00 [9.64, 12.36]
**Subtotal (95% CI)**			**47**			**47**	**44.5%**	**10.95 [9.60, 12.29]**
Heterogeneity: Tau² = 0.00; Chi² = 0.26, df = 1 (P = 0.61); I² = 0%								
Test for overall effect: Z = 15.98 (P < 0.00001)								
**Total (95% CI)**			**93**			**93**	**100.0%**	**6.64 [1.08, 12.19]**
Heterogeneity: Tau² = 28.13; Chi² = 90.36, df = 3 (P < 0.00001); I² = 97%								
Test for overall effect: Z = 2.34 (P = 0.02)								
Test for subgroup differences: Chi² = 5.70, df = 1 (P = 0.02), I² = 82.5%								

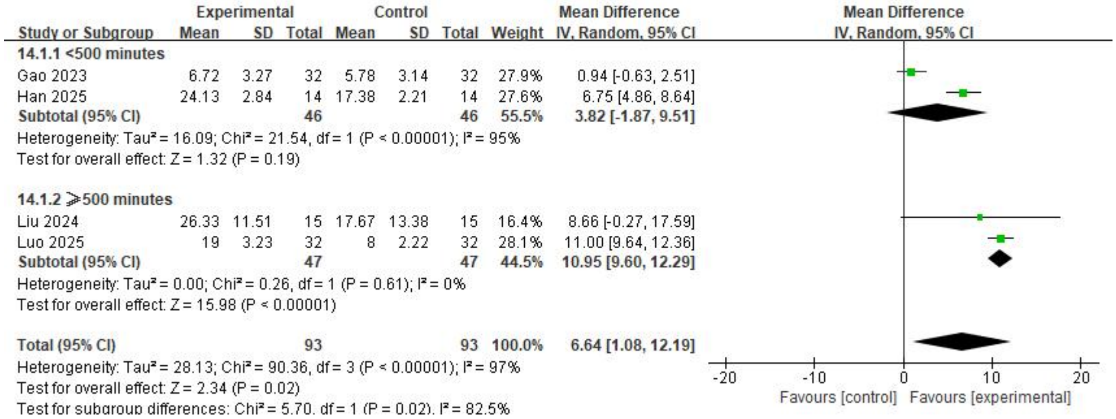

Supplement Figure 15.Forest Plot for the MBI by BCI paradigm

Study or Subgroup	Experimental Mean	Experimental SD	Experimental Total	Control Mean	Control SD	Control Total	Weight	Mean Difference IV, Random, 95% CI
**17.1.1 BCI+Pedaling**								
Gao 2023	6.72	3.27	32	5.78	3.14	32	23.4%	0.94 [-0.63, 2.51]
Han 2025	24.13	2.84	14	17.38	2.21	14	23.2%	6.75 [4.86, 8.64]
**Subtotal (95% CI)**			**46**			**46**	**46.6%**	**3.82 [-1.87, 9.51]**
Heterogeneity: Tau² = 16.09; Chi² = 21.54, df = 1 (P < 0.00001); I² = 95%								
Test for overall effect: Z = 1.32 (P = 0.19)								
**17.1.2 BCI+FES**								
Liu 2024	26.33	11.51	15	17.67	13.38	15	13.5%	8.66 [-0.27, 17.59]
Luo 2025	19	3.23	32	8	2.22	32	23.5%	11.00 [9.64, 12.36]
Sun 2023	33.7	11.09	30	26.53	15.91	30	16.3%	7.17 [0.23, 14.11]
**Subtotal (95% CI)**			**77**			**77**	**53.4%**	**10.81 [9.49, 12.13]**
Heterogeneity: Tau² = 0.00; Chi² = 1.35, df = 2 (P = 0.51); I² = 0%								
Test for overall effect: Z = 16.08 (P < 0.00001)								
**Total (95% CI)**			**123**			**123**	**100.0%**	**6.72 [1.74, 11.69]**
Heterogeneity: Tau² = 26.89; Chi² = 90.37, df = 4 (P < 0.00001); I² = 96%								
Test for overall effect: Z = 2.65 (P = 0.008)								
Test for subgroup differences: Chi² = 5.50, df = 1 (P = 0.02), I² = 81.8%								

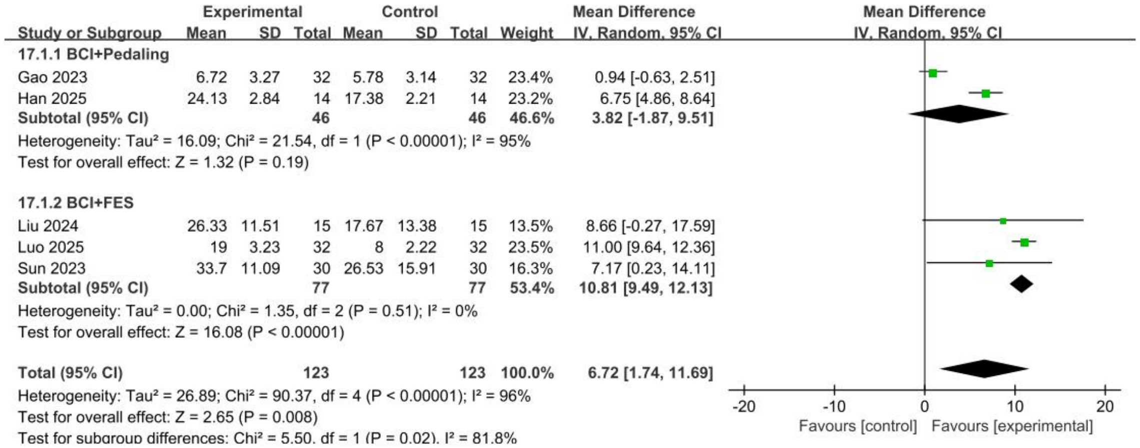

Supplement Table 1.Search strategy in Pubmed

#	Search Query
1	Stroke [MeSH]
2	Strokes OR“Cerebrovascular Accident”OR“Cerebrovascular Accidents”OR CVA (Cerebrovascular Accident) OR CVAs (Cerebrovascular Accident) OR“Cerebrovascular Apoplexy” OR Apoplexy, Cerebrovascular OR “Vascular Accident,” Brain OR “Brain Vascular Accident” OR “Brain Vascular Accidents” OR “Vascular Accidents,” Brain OR “Cerebrovascular Stroke” OR“Cerebrovascular Strokes” OR Stroke, Cerebrovascular OR Strokes, Cerebrovascular OR Apoplexy OR “Cerebral Stroke” OR “Cerebral Strokes” OR Stroke, Cerebral OR Strokes, Cerebral OR Stroke, Acute OR “Acute Stroke” OR“Acute Strokes” OR Strokes, Acute OR “Cerebrovascular Accident,” Acute OR “Acute Cerebrovascular Accident” OR“Acute Cerebrovascular Accidents” OR “Cerebrovascular Accidents,” Acute [Title/Abstract]
3	#1 OR #2
4	"Brain-Computer Interfaces"[Mesh]
5	Brain Computer Interfaces OR Interface, Brain-Computer OR Interfaces, Brain-Computer OR Neural Interface Technology OR Interface Technologies, Neural OR Interface Technology, Neural OR Neural Interface Technologies OR Technologies, Neural Interface OR Technology, Neural Interface OR Brain-Computer Interface OR Brain Computer Interface OR Brain-Machine Interfaces OR Brain-Machine Interface OR Interface, Brain-Machine OR Interfaces, Brain-Machine OR Brain Machine Interface OR Brain Machine Interfaces OR Interface, Brain Machine OR Interfaces, Brain Machine OR Machine Interface, Brain OR Machine Interfaces, Brain[Title/Abstract]
6	#4 OR #5
7	#3 and #6

PRISMA 2020 Checklist

Section and Topic	Item #	Checklist item	Location where item is reported
**TITLE**			
Title	1	Identify the report as a systematic review.	Title
**ABSTRACT**			
Abstract	2	See the PRISMA 2020 for Abstracts checklist.	Abstract
**INTRODUCTION**			
Rationale	3	Describe the rationale for the review in the context of existing knowledge.	Introduction
Objectives	4	Provide an explicit statement of the objective(s) or question(s) the review addresses.	Introduction
**METHODS**			
Eligibility criteria	5	Specify the inclusion and exclusion criteria for the review and how studies were grouped for the syntheses.	Methods – Eligibility criteria
Information sources	6	Specify all databases, registers, websites, organisations, reference lists and other sources searched or consulted to identify studies. Specify the date when each source was last searched or consulted.	Methods – Search strategy
Search strategy	7	Present the full search strategies for all databases, registers and websites, including any filters and limits used.	Methods – Search strategy
Selection process	8	Specify the methods used to decide whether a study met the inclusion criteria of the review, including how many reviewers screened each record and each report retrieved, whether they worked independently, and if applicable, details of automation tools used in the process.	Methods – Eligibility criteria and study selection
Data collection process	9	Specify the methods used to collect data from reports, including how many reviewers collected data from each report, whether they worked independently, any processes for obtaining or confirming data from study investigators, and if applicable, details of automation tools used in the process.	Methods – Data extraction
Data items	10a	List and define all outcomes for which data were sought. Specify whether all results that were compatible with each outcome domain in each study were sought (e.g. for all measures, time points, analyses), and if not, the methods used to decide which results to collect.	Methods – Eligibility criteria
	10b	List and define all other variables for which data were sought (e.g. participant and intervention characteristics, funding sources). Describe any assumptions made about any missing or unclear information.	Methods – Data extraction
Study risk of bias assessment	11	Specify the methods used to assess risk of bias in the included studies, including details of the tool(s) used, how many reviewers assessed each study and whether they worked independently, and if applicable, details of automation tools used in the process.	Methods – Quality assessment
Effect measures	12	Specify for each outcome the effect measure(s) (e.g. risk ratio, mean difference) used in the synthesis or presentation of results.	Methods – Statistical analysis
Synthesis methods	13a	Describe the processes used to decide which studies were eligible for each synthesis (e.g. tabulating the study intervention characteristics and comparing against the planned groups for each synthesis (item #5)).	Methods – Statistical analysis
	13b	Describe any methods required to prepare the data for presentation or synthesis, such as handling of missing summary statistics, or data conversions.	Methods – Data extraction
	13c	Describe any methods used to tabulate or visually display results of individual studies and syntheses.	Methods – Statistical analysis
	13d	Describe any methods used to synthesize results and provide a rationale for the choice(s). If meta-analysis was performed, describe the model(s), method(s) to identify the presence and extent of statistical heterogeneity, and software package(s) used.	Methods – Statistical analysis
	13e	Describe any methods used to explore possible causes of heterogeneity among study results (e.g. subgroup analysis, meta-regression).	Methods – Statistical analysis

PRISMA

PRISMA 2020 Checklist

Section and Topic	Item #	Checklist item	Location where item is reported
	13f	Describe any sensitivity analyses conducted to assess robustness of the synthesized results.	Methods – Statistical analysis
Reporting bias assessment	14	Describe any methods used to assess risk of bias due to missing results in a synthesis (arising from reporting biases).	Methods – Statistical analysis
Certainty assessment	15	Describe any methods used to assess certainty (or confidence) in the body of evidence for an outcome.	Not applicable
**RESULTS**			
Study selection	16a	Describe the results of the search and selection process, from the number of records identified in the search to the number of studies included in the review, ideally using a flow diagram.	Results – Search results
	16b	Cite studies that might appear to meet the inclusion criteria, but which were excluded, and explain why they were excluded.	Results – Search results
Study characteristics	17	Cite each included study and present its characteristics.	Results – Search results / Table 1
Risk of bias in studies	18	Present assessments of risk of bias for each included study.	Results – Table 2
Results of individual studies	19	For all outcomes, present, for each study: (a) summary statistics for each group (where appropriate) and (b) an effect estimate and its precision (e.g. confidence/credible interval), ideally using structured tables or plots.	Results – Effects on Lower-limb motor impairment / Activities of daily living / Figures 2–4
Results of syntheses	20a	For each synthesis, briefly summarise the characteristics and risk of bias among contributing studies.	Results – Subgroup analysis
	20b	Present results of all statistical syntheses conducted. If meta-analysis was done, present for each the summary estimate and its precision (e.g. confidence/credible interval) and measures of statistical heterogeneity. If comparing groups, describe the direction of the effect.	Results – Effects on Lower-limb motor impairment / Activities of daily living
	20c	Present results of all investigations of possible causes of heterogeneity among study results.	Results – Subgroup analysis
	20d	Present results of all sensitivity analyses conducted to assess the robustness of the synthesized results.	Results – Effects on Lower-limb motor impairment / Subgroup analysis
Reporting biases	21	Present assessments of risk of bias due to missing results (arising from reporting biases) for each synthesis assessed.	Results – Publication bias
Certainty of evidence	22	Present assessments of certainty (or confidence) in the body of evidence for each outcome assessed.	Not applicable
**DISCUSSION**			
Discussion	23a	Provide a general interpretation of the results in the context of other evidence.	Discussion
	23b	Discuss any limitations of the evidence included in the review.	Discussion – Limitations

PRISMA

PRISMA 2020 Checklist

Section and Topic	Item #	Checklist item	Location where item is reported
	23c	Discuss any limitations of the review processes used.	Discussion – Limitations
	23d	Discuss implications of the results for practice, policy, and future research.	Discussion – Clinical and research implications
**OTHER INFORMATION**			
Registration and protocol	24a	Provide registration information for the review, including register name and registration number, or state that the review was not registered.	Abstract – Systematic review registration
	24b	Indicate where the review protocol can be accessed, or state that a protocol was not prepared.	Abstract – Systematic review registration
	24c	Describe and explain any amendments to information provided at registration or in the protocol.	Not applicable
Support	25	Describe sources of financial or non-financial support for the review, and the role of the funders or sponsors in the review.	Not applicable
Competing interests	26	Declare any competing interests of review authors.	Conflict of interest
Availability of data, code and other materials	27	Report which of the following are publicly available and where they can be found: template data collection forms; data extracted from included studies; data used for all analyses; analytic code; any other materials used in the review.	Data availability statement

From: Page MJ, McKenzie JE, Bossuyt PM, Boutron I, Hoffmann TC, Mulrow CD, et al. The PRISMA 2020 statement: an updated guideline for reporting systematic reviews. BMJ 2021;372:n71. doi: 10.1136/bmj.n71. This work is licensed under CC BY 4.0. To view a copy of this license, visit https://creativecommons.org/licenses/by/4.0/
